# Impact of Dynasore an Inhibitor of Dynamin II on *Shigella flexneri* Infection

**DOI:** 10.1371/journal.pone.0084975

**Published:** 2013-12-19

**Authors:** Mabel Lum, Stephen R. Attridge, Renato Morona

**Affiliations:** School of Molecular and Biomedical Science, University of Adelaide, Adelaide, South Australia, Australia; Wadsworth Center, New York State Dept. Health, United States of America

## Abstract

*Shigella flexneri* remains a significant human pathogen due to high morbidity among children < 5 years in developing countries. One of the key features of *Shigella* infection is the ability of the bacterium to initiate actin tail polymerisation to disseminate into neighbouring cells. Dynamin II is associated with the old pole of the bacteria that is associated with F-actin tail formation. Dynamin II inhibition with dynasore as well as siRNA knockdown significantly reduced *Shigella* cell to cell spreading *in vitro*. The ocular mouse Sereny model was used to determine if dynasore could delay the progression of *Shigella* infection *in vivo*. While dynasore did not reduce ocular inflammation, it did provide significant protection against weight loss. Therefore dynasore's effects *in vivo* are unlikely to be related to the inhibition of cell spreading observed *in vitro*. We found that dynasore decreased *S. flexneri*-induced HeLa cell death *in vitro* which may explain the protective effect observed *in vivo*. These results suggest the administration of dynasore or a similar compound during *Shigella* infection could be a potential intervention strategy to alleviate disease symptoms.

## Introduction

*Shigella flexneri* is the etiological agent of bacillary dysentery (shigellosis). It is transmitted via the faecal-oral route and is a significant human pathogen due to the high morbidity among children < 5 years in developing countries. Over a period between 1990 - 2009, 125 million shigellosis cases were recorded in Asia, of which ~ 14,000 were fatal [1]. The lack of a vaccine, an increase in multi-drug resistance and the absence of suitable small animal model to study the infection contribute to the persistence of shigellosis [2]. 

The pathogenic determinants of *S. flexneri* are mainly encoded on the large 200 kb virulence plasmid [3]. These proteins are involved in the type three secretion system (TTSS), the modulation of host immune responses, and the mediation of *Shigella* actin-based motility (ABM). *Shigella* bacteria invade the host intestinal epithelium via microfold cells and induce pyroptosis of the resident macrophages in the follicle associated epithelium through caspase-1 activation [4]. Caspase-1 activation releases interleukin-1β (IL-1β) and interleukin-18 (IL-18), resulting in strong inflammatory responses and magnified innate responses, respectively [5]. After *Shigella* bacteria are released into the basolateral compartment, *Shigella* bacteria invade enterocytes via the TTSS, followed by lysis of the endocytic vacuole and replication in the cytoplasm [6,7]. The *Shigella* IcsA protein interacts with the host N-WASP (Neural Wiskott-Aldrich syndrome protein) and Arp2/3 complex to initiate F-actin nucleation and polymerisation, leading to ABM and intracellular spreading and subsequently intercellular spreading via protrusions into adjacent cells. After escaping from the double membrane vacuole, subsequent cycles of infection are initiated [8]. 

*Shigella* ABM is dependent on both the 120 kDa outer membrane protein, IcsA (VirG), and the lipopolysaccharide (LPS) structure [9-11]. IcsA is necessary for pathogenesis as *∆icsA* strains have greatly reduced virulence in human volunteers and in animal infection models [9,12,13]. Smooth *Shigella* strains express the complete LPS molecule, i.e. the lipid A core, core oligosaccharide and O-antigen subunit. In rough strains the O-antigen subunit is absent due to mutations in chromosomal genes encoding for LPS synthesis. Rough strains can invade epithelial cells and initiate ABM but have a defect in intercellular spreading [14,15]. 

Polarised colonic epithelium cells, the site of *Shigella* infection, are characterised by apical junctional complexes (APCs). APCs consist of tight junctions (TJs) and adherens junctions (AJs) at the most apical end, which are undercoated with a prominent network of actin-myosin II (actomyosin) ring [16]. Thus for cell to cell spreading to occur, the tensions of the actomyosin ring have to be overcome before disruption of the cellular contacts can take place [17]. Components of the AJs and TJs such as L-CAM, α-catenin, β-catenin, α-actinin and vinculin are found at the actin tail of *Shigella* during protrusion formation. L-CAM is important in cell to cell spread as it helps to maintain a tight association between the bacterium and the membrane of the protrusions [18,19]. Myosin-X is a component of adherens junctions but are not localised to the *Shigella* actin tail. Knockdown of myosin-X resulted in shortened and thickened protrusion stalks which reduced the bacteria's ability to form plaques [20]. *Shigella* invasion and dissemination is also dependent on ATP release by connexion 26, and formins, Dia1 and Dia2 [21,22]. Similar to the Arp2/3 complex, formins initiate *de novo* actin polymerisation but can also crosslink actin filaments [23]. Recent report suggests *Shigella* preferentially translocate between TJs where three epithelial cells meet, a process dependent on the TJ protein, tricellulin [24]. Bacteria engulfment, but not protrusion formation into the neighbouring cell is triggered by phosphoinositide 3-kinase and is dependent upon dynamin II, Epsin-1 and clathrin which are essential components of the clathrin-mediated endocytic pathway [24,25]. 

Dynamin II is a 96 kDa protein with an N-terminal guanine triphosphatase (GTPase), a middle domain, a pleckstrin homology (PH) domain, a GTPase effector domain (GED) and a C-terminal proline rich domain (PRD) [26-28]. It is a cytoplasmic protein but can be membrane bound via interactions between its PH domain which binds phosphatidylinositol 4,5-bisphosphate [PI(4,5)P_2_] and another region upstream of the PH domain which inserts into the lipid bilayer [29]. Three members of the family have been identified; dynamin I (neurons), dynamin II (ubiquitous) and dynamin III (brain, lung and testis) [30]. Multiple splice variants were also identified for all three dynamin isoforms [30]. In its native state, dynamin is a dimer. In the presence of GTP, helical dimerisation between adjacent dynamin molecules is thought to drive assembly-stimulated GTPase activity, resulting in constriction of the neck of clathrin coated vesicles leading to scission [31-33]. The GTPase activity of dynamin I, dynamin II and the mitochondrial dynamin, Drp1 are inhibited by dynasore, a non-competitive reversible inhibitor [34]. Dynasore has also been shown to have protective effects in various animal models of non-infectious human diseases [35-37].

We hypothesized that *S. flexneri* movement from one cell to the next may depend on various components important for endocytosis and/or exocytosis. Hence we investigated the role of dynamin II in *S. flexneri* cell to cell spread. In this study dynamin II inhibition with dynasore as well as knockdown with dynamin II siRNA reduced *Shigella* plaque formation. We also investigated if dynamin II is important for *Listeria monocytogenes* cell to cell spreading and found that dynamin II inhibition with dynasore reduced *Listeria* plaque formation to a lesser extent. A murine Sereny test was used to determine if dynasore could prevent *Shigella*-induced keratoconjuctivitis. Infected mice treated with the dynasore carrier alone, NMP/PEG, developed a strong inflammatory response and lost significant body weight. Conversely infected mice treated with dynasore lost a significantly smaller portion of body weight but a strong inflammatory response was still observed. If dynasore was inhibiting cell to cell spreading, it is expected that the inflammatory response would be lessened. This prompted us to investigate the effect of dynasore on *S. flexneri*-infected HeLa cells in a cytotoxicity assay. Surprisingly at high dynasore concentrations (≥ 120 μM), HeLa cell death was significantly reduced, which may explain the protective effect observed *in vivo*.

## Materials and Methods

### Bacterial strains and growth media

The strains and plasmids used in this study are listed in Table 1. *Escherichia coli* K-12 strain DH5α was used for routine cloning. All bacterial strains were routinely cultured in Luria Bertani (LB) broth and on LB agar. *S. flexneri* strains were grown from a Congo Red positive colony as described previously [38]. Bacteria were grown in media for 16 h with aeration, subcultured 1/20 and then grown to mid-exponential growth phase by incubation with aeration for 1.5 h at 37°C. Where appropriate, media were supplemented with tetracycline (4 or 10 μg/mL) or kanamycin (50 μg/mL). 

**Table 1:** Bacterial strains and plasmids.

**Strain or plasmid**	**Relevant characteristics^*#*^**	**Reference or source**
**Plasmid**		
pMP7604	Broad host range vector, pME6031 [*mCherry*; Tc^R^]	[39]
***E. coli K-12***		
DH5α	F^-^ φ80*lac*ZΔM15 Δ(lacZYA-argF)U169 recA1 endA1	Gibco-BRL
	hsdR17(rk^-^, mk**^*+*^**) *pho*A*sup*E44 *thi*-1 gyrA96 relA1 λ-	
***S. flexneri***		
2457T	*S. flexneri 2a* wild type	Laboratory collection
MLRM107	2457T [pMP7604; Tc^R^]	This study
RMA723	2457T *ΔrmlD*::Km^R^	[38]
RMA2041	2457T *ΔicsA*::Tet**^*R*^**	[38]
RMA2043	RMA2041 *ΔrmlD*::Km^R^	[38]
RMA2159	Virulence plasmid-cured 2457T	Laboratory collection
***L. monocytogenes***		
DRDC8	Dairy isolate	[70]

^#^ Tet**^*R*^**, Tetracycline resistant; Km^R^, Kanamycin resistant

### DNA methods

Plasmid pMP7604 confers tetracycline resistant and expresses mCherry under the control of the *tac* promoter [39]. mCherry expression was induced with 2 mM IPTG. Strain MLRM107 was constructed by electroporation of pMP7604 into 2457T and selected with LB agar supplemented with tetracycline. DNA manipulation, PCR, transformation and electroporation into *S. flexneri* were performed as previously described [40,41]. 

### Chemicals and antibodies

Dynasore (EC-000.2052; Exclusive Chemistry, D7693-25MG; Sigma-Aldrich, ab120192; Abcam) was prepared as an 80 mM stock in dimethyl sulfoxide (DMSO) (D2650; Sigma-Aldrich) for *in vitro* studies. For *in vivo* studies, dynasore was dissolved in a formulation containing 1-methyl-2-pyrrolidione (NMP/Pharmasolve; Ashland ISP) and polyethylene glycol 300 (PEG300; Sigma-Aldrich) (1 part NMP to 9 parts PEG300). Chemicals were prepared as a 22 mg/mL stock and diluted 1/4 (5.5 mg/mL) with NMP/PEG before injection into mice. Mouse anti-dynamin antibody (610245; BD Biosciences) was used at 1:125 for Western immunoblotting. Rabbit anti-GAPDH antibody (600-401-A33; Rockland Immunochemicals, Inc.) was used at 1:3000 for Western immunoblotting. For immunofluorescence (IF) microscopy, the anti-dynamin antibody, rabbit polyclonal anti-N-WASP [42], Alexa Fluor 488-conjugated donkey anti-rabbit and Alexa 594-conjugated donkey anti-mouse secondary antibody (Molecular Probes) were used at 1:100. 

### Reverse transfection and HeLa cell lysate preparation

DNMII siRNA (L004007-00-0005) and siRNA controls (Non-targeting Pool; D-001810-10-05, siGLO Green Transfection Indicator; D-001630-01-05) were purchased from Thermo Scientific. siRNAs were transfected with DharmaFECT 3 Transfection Reagent (T-2003-03) and DharmaFECT Cell Culture Reagent (DCCR; B-004500-100), also purchased from Thermo Scientific. Reverse transfection of HeLa cells (Human, cervical, epithelial cells ATCC #CCL-70) were carried out based on a method by Thermo Scientific. siRNA were prepared as a 5 µM stock and the final concentration used was 50 nM.

HeLa cells were transfected as follows. 12 µL siRNA, 6 µL DharmaFect 3 and 282 µL DCCR were mixed and transferred to a single well in a 12 well tray (Tray 1) and was incubated for 40 min at RT. For each siRNA, two wells were prepared. After 40 min, 2.5 × 10^5^ HeLa cells in 0.9 mL MEM, 10% FCS were added to each well. The tray was incubated overnight at 37°C, 5% CO_2_. HeLa cells were re-transfected on day 2. 12 µL siRNA, 6 µL DharmaFect 3 and 282 µL DCCR were mixed and transferred to a single well in a 12 well tray (Tray 2) and was incubated for 40 min at RT. HeLa cells from the one well that was transfected the day before (Tray 1) were trypsinised (110 µL 0.1% [w/v] trypsin/0.02% [w/v] EDTA/PBS) and resuspended in 190 µL MEM, 10% FCS. The trypsinised HeLa cells were combined with 600 µL MEM, 10% FCS and were added to the DCCR mixture in Tray 2. The tray was incubated overnight at 37°C, 5% CO_2_. On day 3, HeLa cell lysate extracts were prepared as described by Qualmann and Kelly [43] with some modifications. Re-transfected HeLa monolayers from two wells of a 12-well tray were washed with PBS for 15 - 20 min, dislodged with 110 μL trypsin and resuspended in 190 μL PBS. The cells were pelleted by centrifugation (3,500 rpm, 5 min, RT, Eppendorf 5415R) and the pellet was resuspended in 80 μL in 0.1 % (v/v) Triton X-100 in lysis buffer A (10 mM HEPES pH 7.4, 150 mM NaCl, 1 mM EGTA, 0.1 mM MgCl_2_) supplemented with protease inhibitors (5 μg/mL pepstatin, 10 μg/mL aprotinin, 10 μg/mL chymostatin, 1 mM PMSF). The cells were lysed at 4°C for 30 min and were pelleted by centrifugation (25,000 rpm, 30 min, 4°C, Beckman OptimaTM MAX-UP Ultracentrifuge). The supernatant was collected and mixed with 80 μL 2× Sample buffer (0.125 M Tris-HCl, pH 6.8, 4% [w/v] SDS, 20% [v/v] glycerol, 10% [v/v] β-mercaptoethanol, 0.04% [w/v] Bromophenol blue), and stored at -20°C. Samples were heated at 100°C for 5 min prior to SDS-PAGE. 

### SDS-PAGE and Western immunoblotting

SDS-PAGE (12% acrylamide gel) and Western immunoblotting were carried out as described previously [42]. Molecular weight markers used were BenchMark™ Pre-Stained Protein Ladder (Invitrogen).

### Plaque assay

Plaque assays were performed with HeLa cells as described previously by Oaks et al. [44] with modifications. 1.2 × 10^6^ HeLa cells were seeded in six-well trays in minimal essential medium (MEM), 10% FCS, 1% penicillin/streptomycin. Cells were grown to confluence overnight and were washed twice with Dulbecco’s modified Eagle medium (DMEM) prior to inoculation. 2.5 × 10^4^ mid-exponential phase bacteria were added to each well. Trays were incubated at 37°C, 5% CO_2_ and were rocked gently every 15 min to spread the inoculum evenly across the well. At 90 min post infection, the inoculum was aspirated. 3 mL of the first overlay (DMEM, 5% FCS, 20 µg/mL gentamicin, 0.5% (w/v) agarose [Seakem ME]) was added to each well. Dynasore or DMSO were added and were swirled to ensure even distribution. The second overlay (DMEM, 5% FCS, 20 µg/mL gentamicin, 0.5% (w/v) agarose, 0.1% (w/v) Neutral Red solution [Gibco BRL]) was added 48 h post infection and plaques were imaged 6 h later. All observable plaques were counted and the diameter was measured for each condition in each experiment. At least 25 plaques were measured for each condition.

### Infection foci assay

HeLa cells were transfected prior to infection foci assay as per “***Reversetransfection***”. On day 3, the infection foci assay was carried out. Transfected HeLa cells were washed twice with DMEM prior to inoculation. 5 × 10^4^ mid-exponential phase bacteria expressing mCherry were added to each well. Trays were incubated at 37°C, 5% CO_2_ and were rocked every 15 min to spread the inoculum evenly across the well. At 90 min post infection, the inoculum was aspirated. 1.5 mL of DMEM (phenol red-free) (31053-028; Life Technologies), 1 mM sodium pyruvate, 5% FCS, 20 µg/mL gentamicin, 2 mM IPTG was added to each well. 24 h later the infection foci were imaged with an Olympus IX-70 microscope using a 10× objective. The filter set used was DA/FI/TX-3X-A-OMF (Semrock). Fluorescence and phase contrast images were captured and false colour merged with the Metamorph software program (Version 7.7.3.0, Molecular Devices). The area of the infection foci i.e. area where mCherry was expressed, was outlined and measured with Metamorph. All observable infectious foci were counted and the area was measured for each condition in each experiment. At least 20 infectious foci were measured for each condition.

### Invasion assay and IF microscopy

HeLa cells (8 × 10^4^) were seeded onto sterile glass cover slips in 24-well trays in MEM, 10% FCS, 1% penicillin/streptomycin. For transfected HeLa cells, refer to “***Reversetransfection***”. The following modifications were made. On day 1, one well was prepared for each siRNA. On day 2, the siRNA:DharmaFect3:DCCR mixture was halved. 80% of the HeLa cells that had been transfected on day 1 were reseeded in Tray 2. Cells were grown to semi-confluence overnight, washed twice with Dulbecco’s PBS (D-PBS) and once with MEM, 10% FCS. 4 x 10^7^ mid-exponential phase bacteria were added to each well and subsequently centrifuged (2,000 rpm, 7 min, Heraeus Labofuge 400 R) onto HeLa cells. After 1 h incubation at 37°C, 5% CO_2_, the infected cells were washed thrice with D-PBS and incubated with 0.5 mL MEM containing 40 µg/mL of gentamicin for a further 1.5 h. Infected cells were washed thrice in D-PBS, fixed in 3.7% (v/v) formalin for 15 min, incubated with 50 mM NH_4_Cl in D-PBS for 10 min, followed by permeabilization with 0.1% Triton X-100 (v/v) for 5 min. After blocking in 10% FCS in PBS, the infected cells were incubated at 37°C for 30 min with the desired primary antibody. After washing in PBS, coverslips were incubated with either Alexa Fluor 594-conjugated donkey anti-rabbit or Alexa 594-conjugated donkey anti-mouse secondary antibody (Molecular Probes) (1:100). F-actin was visualised by staining with Alexa Fluor 488-conjugated phalloidin (2 U/mL) and 4’,6’-diamidino-2-phenylindole (DAPI) (10 μg/mL) was used to counterstain bacteria and HeLa cell nuclei. Coverslips were mounted on glass slides with Mowiol 4-88 (Calbiochem) containing 1 µg/mL *p*-phenylenediamine (Sigma) and was imaged using a 100× oil immersion objective (Olympus IX-70). The filter set used was DA/FI/TX-3X-A-OMF (Semrock). Fluorescence and phase contrast images were false colour merged using the Metamorph software program. 

### Protrusion formation

HeLa cells were seeded, infected and fixed as per "Invasion assay and IF microscopy". HeLa cells were washed twice with 1× Annexin V binding buffer (99902; Biotium) prepared in milliQ (18.2 MΩ·cm) water, mounted on glass slides with the same buffer and were imaged using a 40× oil immersion objective (Olympus IX-70). Protrusion formation was defined as any extensions of bacterial projection(s) (minimum of a full bacterial length) beyond the periphery of the HeLa cell. For each condition in each experiment, a minimum of 100 cells were imaged.

### Assay for growth of intracellular bacteria

HeLa cells (8 × 10^4^) were seeded in 24-well trays in MEM, 10% FCS, 1% penicillin/streptomycin. Cells were grown to semi-confluence overnight, washed twice with Dulbecco’s PBS (D-PBS) and once with MEM, 10% FCS. 4 × 10^7^ mid-exponential phase bacteria were added to each well (multiplicity of infection, MOI ~ 500). The bacteria were centrifuged (2,000 rpm, 7 min, Heraeus Labofuge 400 R) onto HeLa cells. After 1 h incubation at 37°C, 5% CO_2_, the infected cells were washed thrice with D-PBS and incubated with 0.5 mL MEM containing 40 µg/mL of gentamicin. At the indicated intervals, monolayers (in duplicate) were washed four times in D-PBS and were lysed with 0.1% (v/v) Triton X-100 in PBS for 5 min and bacteria were enumerated on tryptic soy agar (TSA; Gibco) plates. 

### LDH cytotoxicity assay

HeLa cells (3 × 10^4^) were seeded in 96-well trays in MEM, 10% FCS, 1% penicillin/streptomycin. Cells were grown to confluence overnight and were washed twice with PBS. 50 μL phenol-red free DMEM (31053-028; Life Technologies), 1 mM sodium pyruvate and 3 × 10^7^ mid-exponential phase bacteria ( MOI ~ 1000) in 50 μL PBS or PBS were added into each well, where appropriate. The bacteria were centrifuged (2,000 rpm, 7 min, Heraeus Labofuge 400 R) onto HeLa cells. After 1 h incubation at 37°C, 5% CO_2_, the infected cells were washed thrice with PBS and incubated with 0.1 mL phenol-red free MEM, 40 µg/mL of gentamicin for 4 h. LDH activity was measured with the Cytotoxicity Detection Kit^Plus^ as per manufacturer's instructions (Roche). The percentage of LDH released was calculated with the following formula: ((experimental LDH release − spontaneous LDH release)/(maximal LDH - spontaneous LDH release)) × 100. 

### Ethics statement

The use of animals in this study has been approved by the University of Adelaide Animal Ethics Committee (Project number: S-2012-90). All animals used were handled in compliance with the Australian code of practice for the care and use of animals for scientific purposes, 7th edition (2004). 

### Mouse Sereny test

The mouse Sereny test [45] was carried out as follows. Male Balb/c mice (20-22g) were inoculated with 2.5 × 10^7^ or 5 × 10^8^ CFUs bacteria in 5 μL of bacterial suspension (in PBS) into the right eye; the left eye served as a diluent control. To ascertain the impact of dynasore on ocular infection, mice were injected intraperitoneal (IP) with drug at a dose rate of 30 mg/kg, at t = -1, +6, +23 and +30 hours with respect to infection at 0 hours. Keratoconjunctivitis was evaluated at specific time points after inoculation and scored on a scale ranging from 0 (no infection), 1 (mild keratoconjuctivitis where the eye lid is slightly swollen), 2 (severe keratoconjuctivitis where the eye is half closed) and 3 (fully developed keratoconjunctivitis where the eye is completely closed). 

Ethics concerns regarding the *in vivo* use of DMSO prompted us to adopt a different vehicle, NMP/PEG, which is made up of 1 part 1-methyl-2-pyrrolidione (NMP) and 9 parts polyethylene glycol 300 (PEG300), for *in vivo* studies designed to assess the impact of dynasore in this model. *In vitro* studies showed that plaque formation by *S. flexneri* strain 2457T was comparable using either diluent (Figures S1A and S1B). A preliminary mouse study showed that for injections of dynasore (Figure S1C) or vehicle (Figure S1D) resulted in comparable (~5%) weight loss. 

### Sectioning and H&E staining of mouse eyes and eyelids

For histopathological observations, the infected and uninfected eyes (together with eyelids) were removed and fixed in 4% buffered formaldehyde overnight before embeddeding in paraffin. Sections were prepared and stained with hematoxylin plus eosin by the Ophthalmic Research Laboratories, Centre for Neurological Diseases, Adelaide, Australia.

### Statistical analysis

Statistical analysis was carried out using GraphPad Prism 6. Results are expressed as means ± SEM of data obtained in independent experiments. Statistical differences between two groups were determined with a two-tailed unpaired *t*-test. Statistical differences between three or more groups were determined with a one-way ANOVA followed by Tukey's or Dunnett's multi comparison post hoc test. Statistical significance was set at *p* < 0.05. 

## Results

### Dynamin II is important for *S. flexneri* cell to cell spreading but not protrusion formation

**Figure 1:**
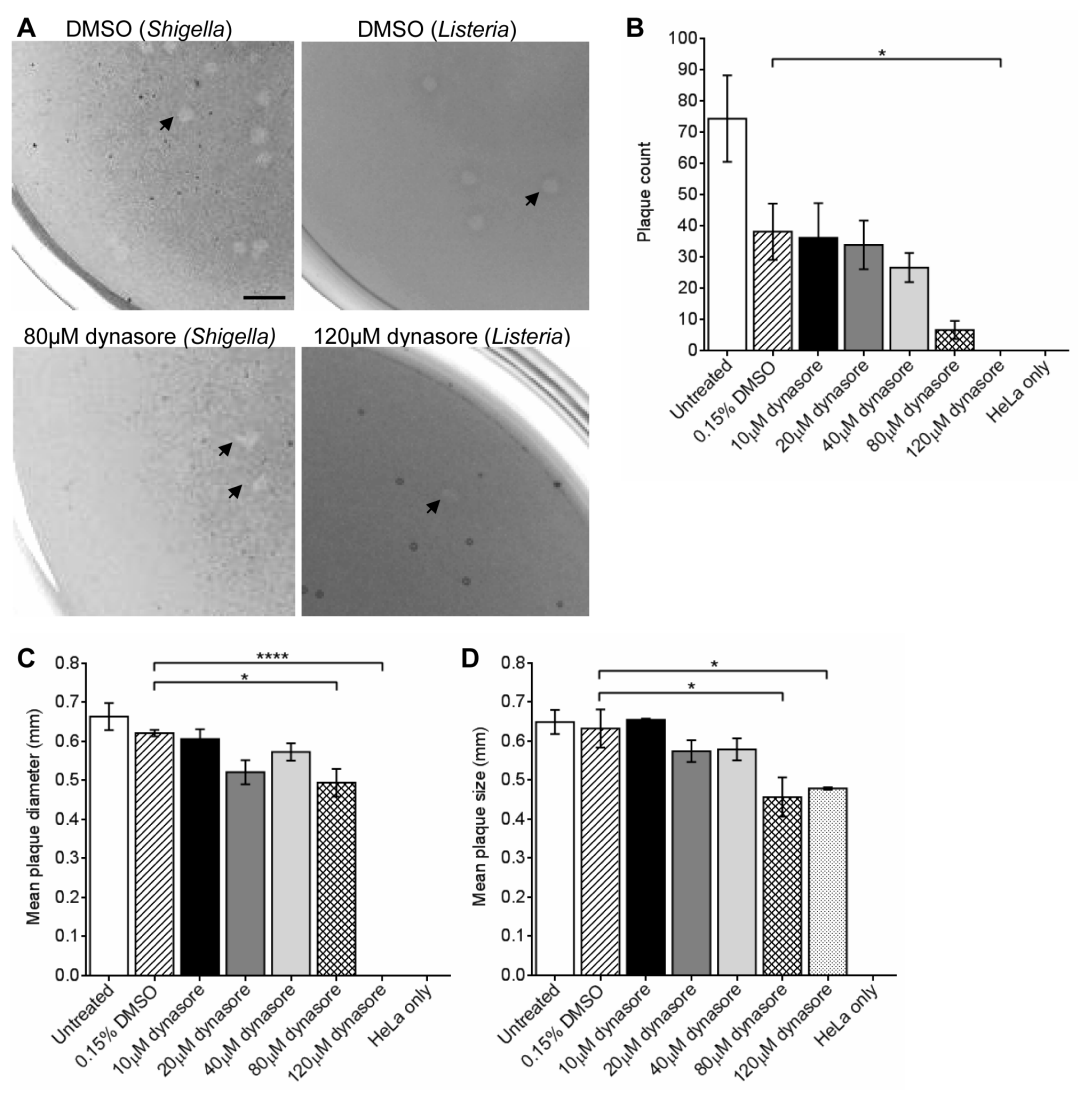
Dynamin II inhibition by dynasore reduces *S. flexneri* 2457T plaque counts and plaque size. HeLa cells were infected with either *S. flexneri* 2457T or *Listeria* in a plaque assay using a 6-well tray as described in the Methods. Plaque formation was performed in the presence of increasing concentrations of dynasore or the vehicle, 0.15% DMSO. (A) Wells were stained with Neutral Red to makes plaques more visible. Scale bar = 2 mm. (B) The total plaque counts or (C) mean plaque diameters from each well infected with *Shigella* were calculated. (D) Mean plaque diameters from each well infected with *Listeria* were calculated. Data are represented as mean ± SEM of independent experiments (n = 4 for *Shigella* and n = 3 for *Listeria*), analysed with one-way ANOVA (*p* < 0.0001), followed by Tukey's post hoc test (*p < 0.05, ****p < 0.0001).

We investigated if the process of cell to cell spreading was dependent on dynamin II, a component of endocytosis. The effects of dynasore, a dynamin II inhibitor, and dynamin II siRNA on plaque formation by *S. flexneri* were determined. HeLa monolayers infected with *S. flexneri* were treated with increasing concentrations of dynasore or with the DMSO vehicle alone. Treatment with 80 µM dynasore reduced plaque diameter (*p < 0.05) and addition of 120 µM dynasore abolished plaque formation altogether (Figure 1A - 1C). *S. flexneri* entry into HeLa cells and intracellular growth were not affected (Figures S2 - S3). We also investigated if dynamin II is important for *Listeria* cell to cell spreading. HeLa monolayers infected with *Listeria* were treated with the DMSO control or increasing concentrations of dynasore. The plaque diameter of *Listeria*-infected HeLa cells treated with 80 and 120 µM dynasore were significantly reduced (*p < 0.05) (Figure 1A and 1D). No difference in plaque diameter was observed between 80 µM and 120 µM dynasore.

**Figure 2:**
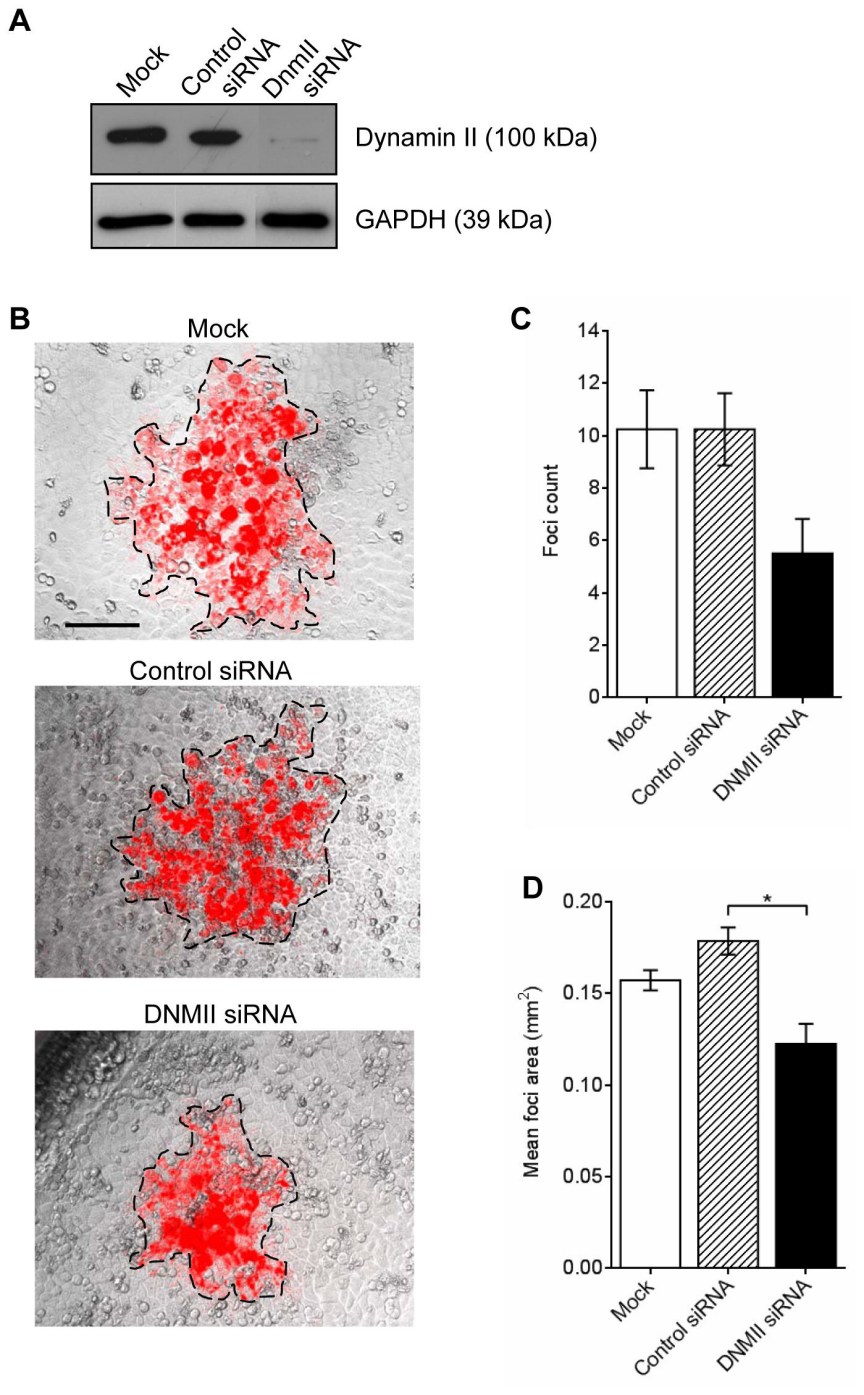
Transfection of HeLa cells with dynamin II siRNA reduces *S. flexneri* MLRM107 foci counts and foci area. HeLa cells were either mock transfected or transfected with control or dynamin II siRNA for 24 h, trypsinised and re-transfected for further 24 h. (A) HeLa cell extracts were probed with anti-dynamin. GAPDH was used as a loading control. (B - D) Post transfection, HeLa cells were infected with *S. flexneri* MLRM107 in an infectious foci assay using a 12-well tray as described in the Methods. Infection foci were imaged 24 h post gentamicin treatment. Images shown are overlay of an image taken with phase contrast and TxRed filter (10× magnification). The area of the infection foci i.e. area where mCherry was expressed, is outlined. Scale bar = 0.1 mm. (C) The total foci counts from one well or (D) mean foci area from one well were calculated. Data are represented as mean ± SEM of independent experiments (n=3). For total foci count analysed with one-way ANOVA, *p* > 0.05. Mean foci area was analysed with one-way ANOVA (*p* = 0.0027), followed by Tukey's post hoc test (*p < 0.05).

To investigate the effect of dynamin II depletion on *S. flexneri* cell to cell spreading, HeLa cells were transfected with dynamin II siRNA and an infectious foci assay was carried out. Western immunoblots of HeLa cell lysates two days post siRNA treatment showed ~95% reduction in dynamin II levels (Figure 2A). *S. flexneri* formed infectious foci on HeLa cells treated with dynamin II siRNA with a reduced foci area but not foci counts when compared to HeLa cells treated with the negative control siRNA (*p < 0.05) (Figure 2C - 2D). Therefore dynamin II inhibition with dynasore as well as siRNA knockdown reduced *Shigella* cell to cell spreading. 

**Figure 3:**
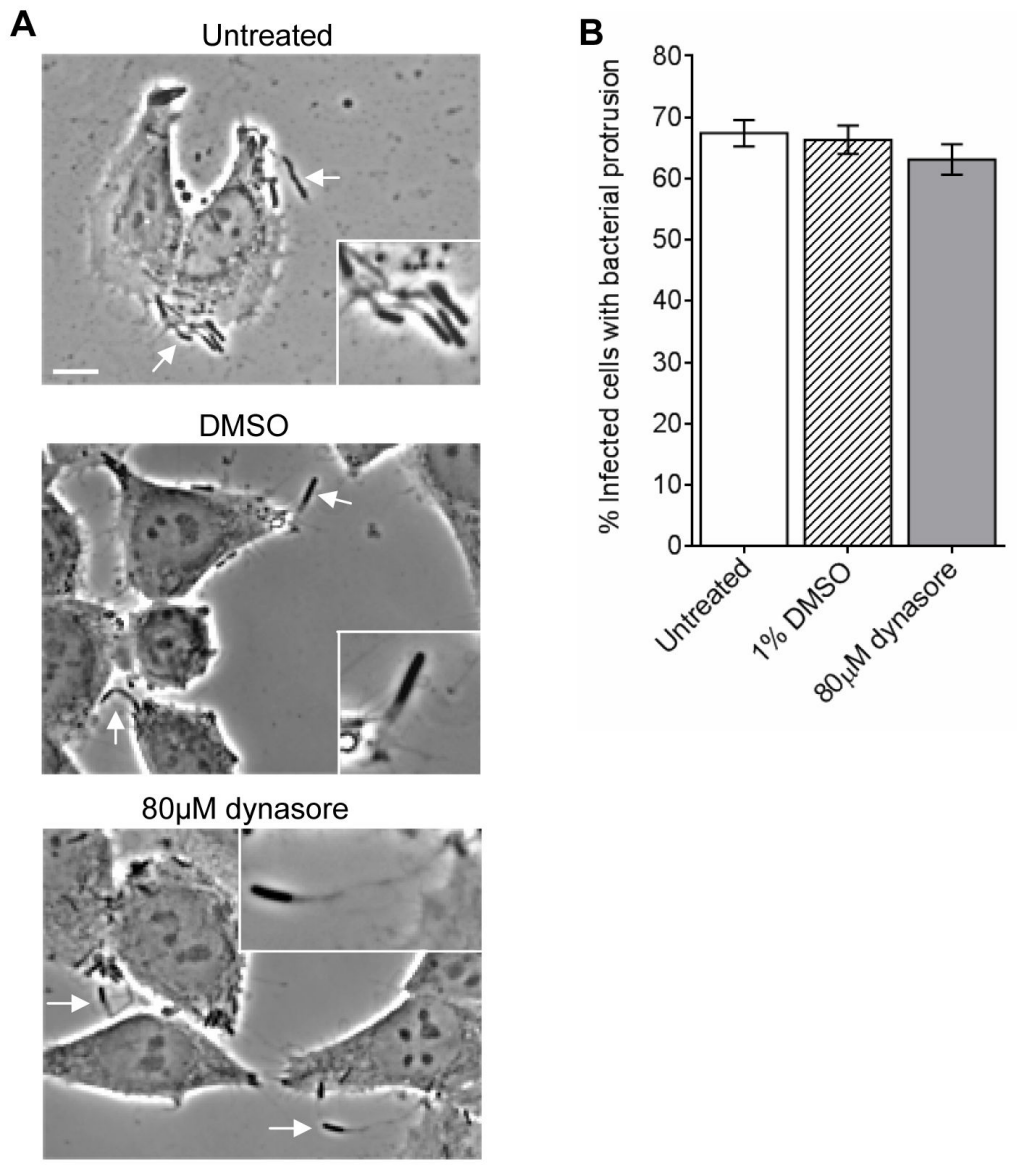
*S. flexneri* 2457T protrusion is not affected in the presence of dynasore. HeLa cells were infected with *S. flexneri* 2457T for 1 h in a 24-well tray. HeLa cells were washed thrice with D-PBS and incubated with MEM containing 40 µg/mL of gentamicin (t=0) to exclude extracellular bacteria. Concurrently HeLa cells were treated with 80 μM dynasore or DMSO for 1.5 h. At t = 1.5, HeLa cells were fixed to observe bacteria protrusions. (A) Infected HeLa cells were imaged at 40× magnification. Scale bar = 10 μm. The arrows indicate protrusion formation. Insert shows 2× enlargement of the indicated region. (B) The percentage of infected cells with bacteria protrusion(s) were enumerated by counting >100 cells in three independent experiments. Data are represented as mean ± SEM of independent experiments (n = 3), analysed with one-way ANOVA (*p* > 0.05).

Next we investigated if dynamin II is involved *S. flexneri* protrusion formation, which may account for the reduction in plaque formation. HeLa monolayers infected with *S. flexneri* were treated with the DMSO control or 80 μM dynasore and the percentage of infected cells with one or more bacteria protrusions were enumerated by counting >100 cells in each experiment (Figure 3A and 3B). 66.33 ± 1.35% of infected HeLa cells had one or more protrusions. Protrusion formation was not affected by the DMSO control (67.42 ± 1.25%) or 80 μM dynasore (63.12 ± 1.43%). It should be noted that while our assessment of the effect of dynasore on plaque formation used confluent cells, and its effect on protrusion formation used semi confluent cells; this approach to study cell to cell spreading has previously been reported by others [20,22,46-49]. 

### Dynamin II is localised to the F-actin tail and protrusions of *S. flexneri*, adjacent to N-WASP

**Figure 4:**
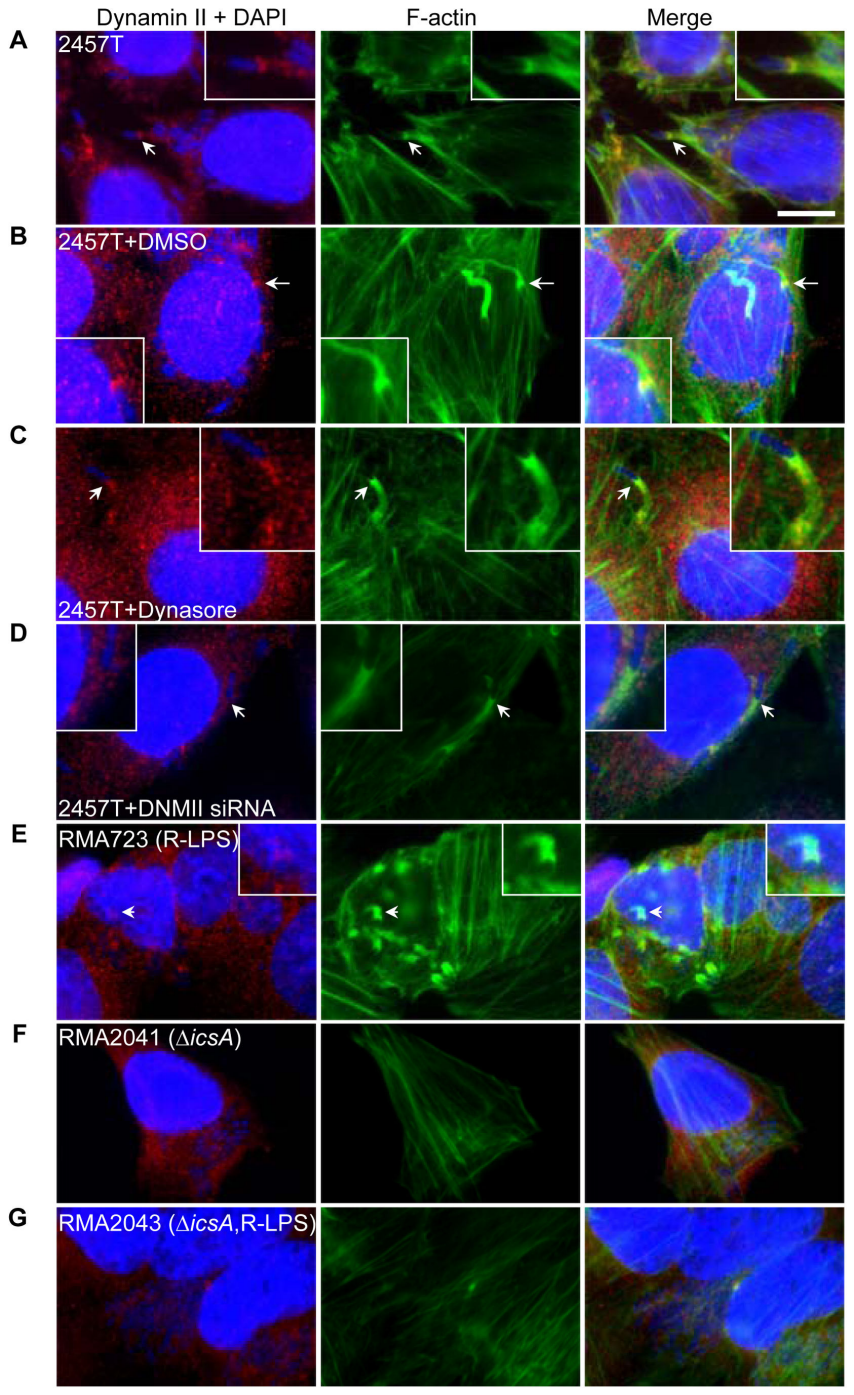
Dynamin II is localised to the S. *flexneri* 2457T F-actin tails and protrusions. HeLa cells were infected with *S. flexneri* 2457T in an invasion assay as described in the Methods. Bacteria and HeLa nuclei were stained with DAPI (blue), F-actin was stained with FITC-phalloidin (green) and dynamin II was stained with anti-dynamin and Alexa Fluor 594-conjugated secondary antibody (red). Images were taken at 100× magnification. Scale bar = 10 μm. (A) – (D) HeLa cells were treated with DMSO, dynasore or were transfected with dynamin II siRNA and were infected with *S. flexneri* 2457T; (A) Untreated; (B) 1% DMSO; (C) 80 μM dynasore; (D) Dynamin II siRNA transfected HeLa cells. (E) – (G) HeLa cells were infected and treated as above with *S. flexneri* control stains; (E) RMA723 (R-LPS - *∆rmlD*); (F) RMA2041 (∆icsA); (G) RMA2043 (∆icsA ∆rmlD). The arrows indicate dynamin II localisation at comet tails or protrusions. Insert shows 2× enlargement of the indicated region. The experiment was repeated twice and representative images are shown.

The polarly localised *S. flexneri* IcsA protein interacts with host proteins such as N-WASP and Arp2/3 complex to initiate actin polymerisation to form an actin tail which imparts motility to the bacteria [50-52]. We investigated dynamin II localisation in *S. flexneri* infected HeLa cells by IF microscopy. Dynamin II was localised to the S. *flexneri* F-actin tail and/or protrusion (Figure 4A). F-actin labelling with FITC-phalloidin does not readily distinguish F-actin tails from protrusion formation as both architectures appear as long F-actin structures behind one pole of the bacteria. We observed 109 infected HeLa cells, and found that 150 out of 158 (95%) of *S. flexneri* F-actin tails and/or protrusions were localised with dynamin II. Treatment with DMSO and 80 µM dynasore did not affect dynamin II localisation (Figure 4B and 4C). In 104 infected HeLa cells treated with 80 µM dynasore, 143 out of 154 *S. flexneri* (93%) F-actin tails and/or protrusions were localised with dynamin II. Similarly in 109 infected HeLa cells treated with the DMSO control, 152 out of 159 *S. flexneri* (96%) F-actin tails and/or protrusions were localised with dynamin II. These results suggest dynamin II interaction at the S. *flexneri* F-actin tail and/or protrusion does not require the activity of its GTPase domain. In dynamin II siRNA transfected cells, a reduction in cellular dynamin II protein levels was observed, as expected. Reduction of dynamin II protein level did not affect dynamin II localisation to the bacteria F-actin tail (Figure 4D). 

In R-LPS strains, IcsA polar localisation and ABM is affected but the bacteria can still invade the cells [53]. The R-LPS strain can form F-actin tails, albeit infrequently, but these are shortened and distorted [54]. Similarly *S. flexneri icsA* mutants in either the smooth or rough LPS background can invade HeLa cells. In the R-LPS strain dynamin II is recruited to *S. flexneri* F-actin tail (Figure 4E) but no recruitment was observed for the *∆icsA* mutant strains (Figure 4F and 4G). This suggests that dynamin II may interact with the F-actin tail since F-actin *de novo* synthesis is dependent on IcsA. Alternately dynamin II could also be interacting indirectly with the F-actin tail through other proteins such as N-WASP which are found at the S. *flexneri* bacterial surface [55]. HeLa cells infected with *S. flexneri* were labelled with anti-N-WASP and anti-dynamin (Figure S4). N-WASP is located immediately at the pole of *S. flexneri* (Figure S4A), forming a cap. Dynamin II labelling also resembles a cap at the S. *flexneri* pole (Figure S4B) and is adjacent to the N-WASP cap with a seemingly small area of overlap (Figure S4C - S4D).

### Effect of dynasore on *S. flexneri* infection of mice

We next investigated the possible *in vivo* relevance of the observed association between dynamin II and *S. flexneri* spreading. A mouse Sereny test was established and used to determine whether ocular infection by *S. flexneri* could be inhibited by the administration of dynasore. In initial studies, mice were infected with 5 × 10^8^ CFUs WT *S. flexneri* 2457T in the right eye and the left eye was used as the control (Figure S5A). At 24, 48 and 72 h, scores between 0 and 3 were given depending on the severity of the inflammation. Histology was carried out to compare sections prepared from eyes with scores of 0 (left eye PBS control) and 3 (fully developed keratoconjuctivitis). No inflammation was observed as expected in the PBS control (Figure S5B, left image). In the *Shigella* infected eye, desquamation and degeneration of the palpebral conjunctiva and fornix epithelial were observed as well as infiltration of polymorphonuclear leukocytes into the epithelial layers (Figure S5B, right image). 

Mice were infected with WT, *∆icsA* or virulence plasmid negative (VP¯) *S. flexneri* strains which are virulent, attenuated or are completely avirulent, respectively, to establish suitable controls. Mice infected with the *∆icsA* or VP¯ strains did not develop any observable inflammation (Figure S5C). Mice infected with WT *S. flexneri* developed a strong inflammatory response with a score of ≥ 2.5. The inflammation peaked at 24 and 30 h with four out of six mice developing strong inflammatory reaction. Over the next two days, the inflammation slowly resolved with two out of six mice developing a strong inflammatory response (score of 3) (Figure S5D). Mice were also weighed on D0 and D3 of the experiment. Mice infected with the avirulent VP¯ strain did not lose weight, however mice infected with either WT or *∆icsA* strains had comparable weight loss (~ 6-9%) (Figure S5E). 

**Figure 5:**
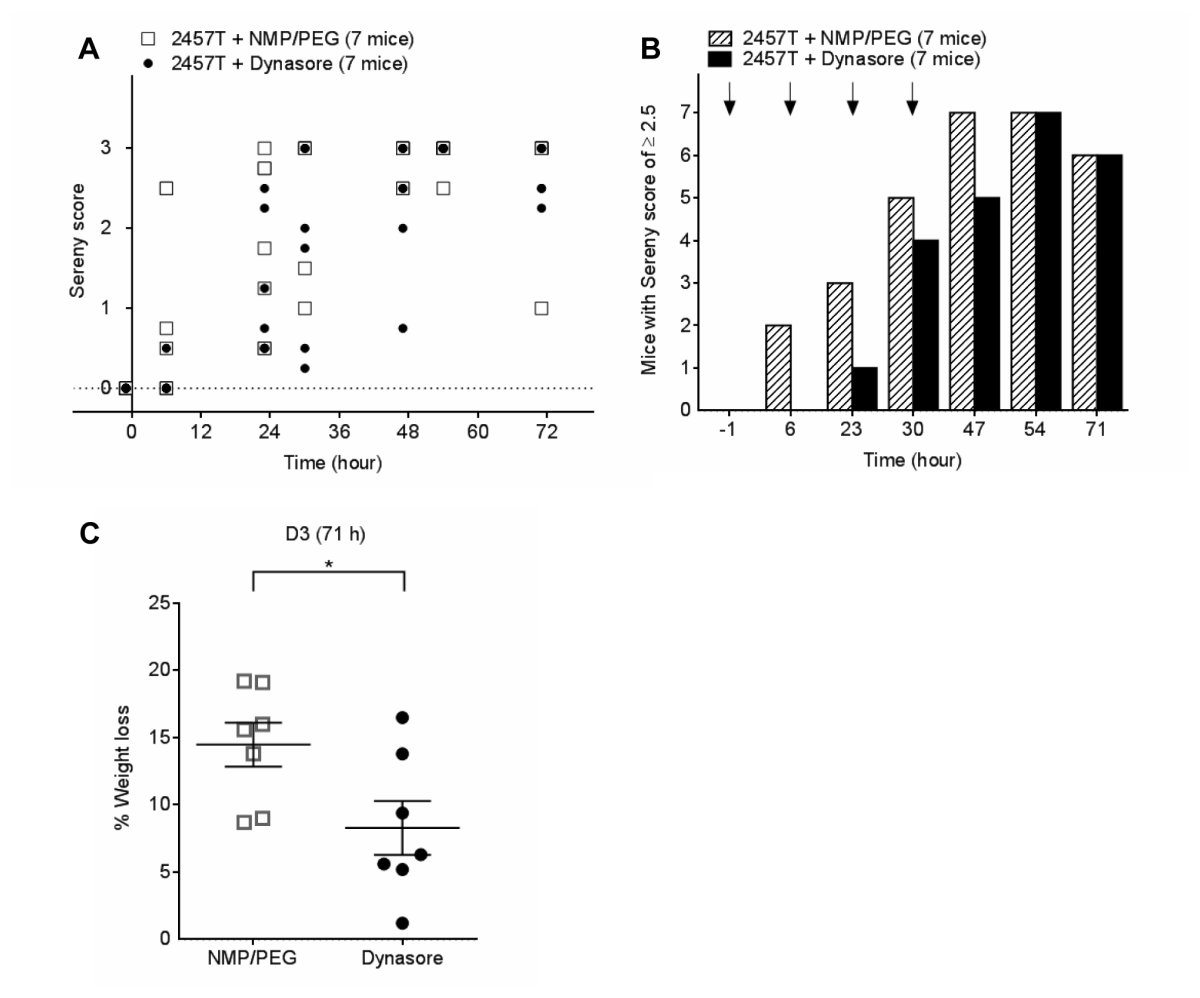
Dynasore protects mice against weight loss resulting from *S. flexneri* 2457T ocular infection (Experiment 1). (A) Sereny scores of mice infected with 5 × 10^8^ CFUs *S. flexneri* 2457T at t = 0 and IP injected with either 100 μL of 5.5 mg/mL of dynasore in NMP/PEG (30mg/kg) or 100 μL NMP/PEG at t = -1, 7, 23, 30 h post inoculation. Each symbol represents one or more mice. (B) The total number of mice with a Sereny score of ≥ 2.5 was calculated and plotted as a bar graph for each time point. Arrows indicate injection with dynasore or NMP/PEG alone. (C) The percentage weight loss for each mouse on D3 was calculated. Data are represented as mean ± SEM (Student's *t*-test, *p < 0.05).

In the first *in vivo* study with dynasore, mice were infected with 5 × 10^8^ CFUs *S. flexneri* 2457T in the right eye (t = 0) and were injected IP with dynasore (30 mg/kg) or vehicle at t = -1, 6, 23 and 30 h. The mice in both groups developed ocular inflammation at a similar rate and the Sereny scores were similar (Figure 5A and 5B). Mice in both groups also had ruffled fur and were often reluctant to move, suggesting that the combination of *Shigella* infection and injection of NMP/PEG (alone or in combination with dynasore) was detrimental to the mice. Mice injected with NMP/PEG lost 15% of their weight during the course of the infection, which was significantly higher than that observed in infected mice not receiving injections (Figure S5E) or in uninfected mice injected with the vehicle or dynasore (Figures S1C and S1D). Mice injected with vehicle only also lost more weight (14.49 ± 1.63%) compared to mice injected with dynasore (8.29 ± 2.01%) (*p < 0.05) (Figure 5C). 

**Figure 6:**
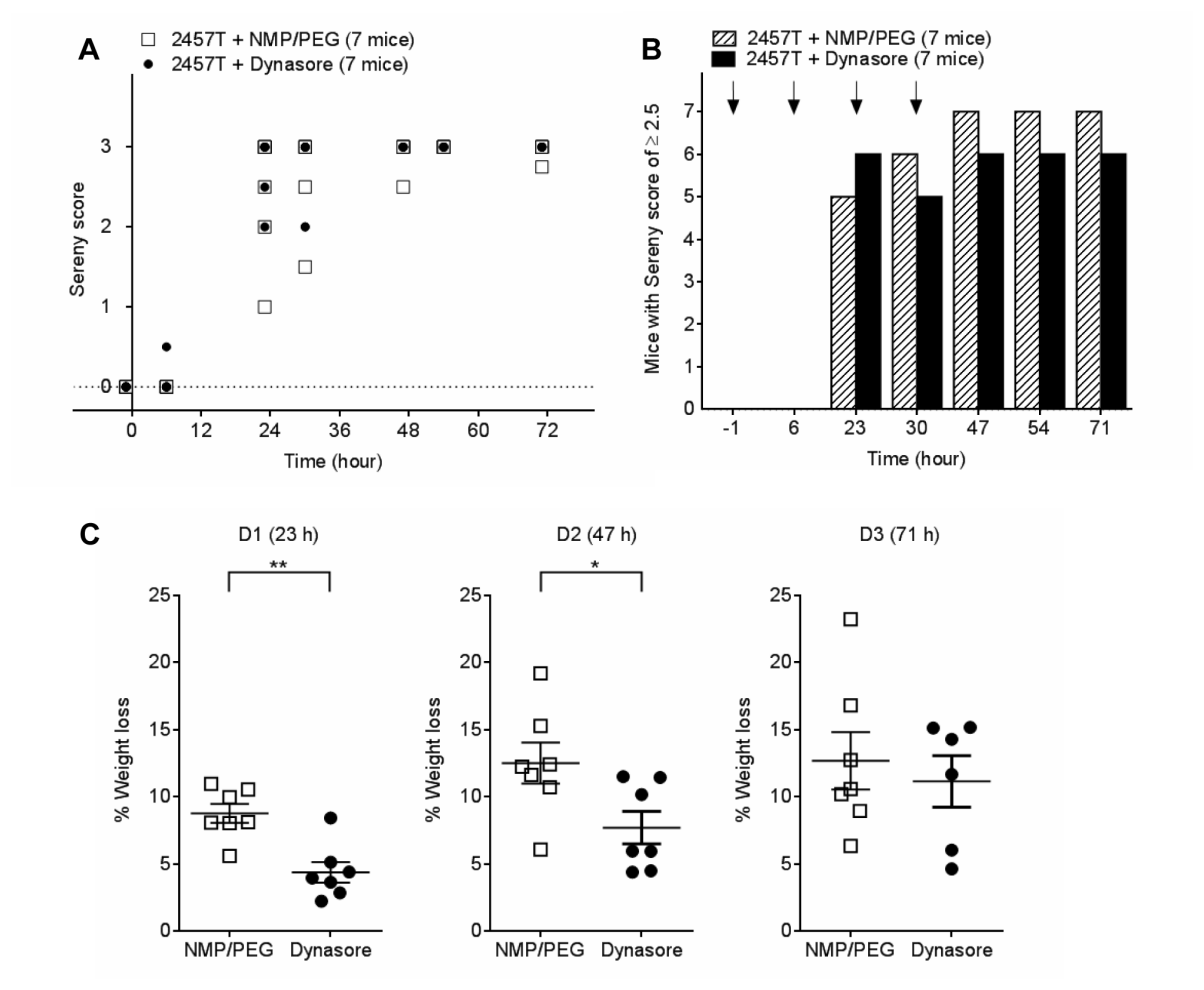
Dynasore protects mice against weight loss resulting from *S. flexneri* 2457T ocular infection (Experiment 2). (A) Sereny scores of mice infected with 5 × 10^8^ CFUs *S. flexneri* 2457T at t = 0 and IP injected with either 100 μL of 5.5 mg/mL of dynasore in NMP/PEG (30mg/kg) or 100 μL NMP/PEG at t = -1, 7, 23, 30 h post inoculation. Each symbol represents one or more mice. (B) The total number of mice with a Sereny score of ≥ 2.5 was calculated and plotted as a bar graph for each time point. Arrows indicate injection with dynasore or NMP/PEG alone. (C) The percentage weight loss for each mouse on D1, D2 and D3 were calculated. Data are represented as mean ± SEM (Student's *t*-test, *p < 0.05, **p < 0.01).

The experiment was repeated with a second group of mice since the first group had varied Sereny scores at respective time points. Mice were infected with 5 × 10^8^ CFUs *S. flexneri* 2457T in the right eye (t = 0) and were IP injected with dynasore (30 mg/kg) or the vehicle at t = -1, 6, 23 and 30 h and were weighed daily. Mice developed the ocular inflammation at the same rate and there was no difference in Sereny scores (Figure 6A and 6B). Mice injected with dynasore lost significantly less weight compared to mice treated with the vehicle alone on D1 (4.39 ± 0.76% vs. 8.77 ± 0.71%, **p < 0.01) and D2 (7.71 ± 1.21% vs. 12.52 ± 1.53%, *p < 0.05) (Figure 6C). Weight loss which was previously observed on D3 in the first experiment (Figure 5C) was not observed in the second group of mice. The latter group of mice collectively had less variations in their Sereny scores (Figure 6A), which could explain why the weight differences were only observed on D1 and D2. Nonetheless these studies suggest that dynasore provided a protective effect against weight loss associated with *S. flexneri* infection.

### Effect of dynasore on mice infected with a low inoculum of *S. flexneri* 2457T

**Figure 7:**
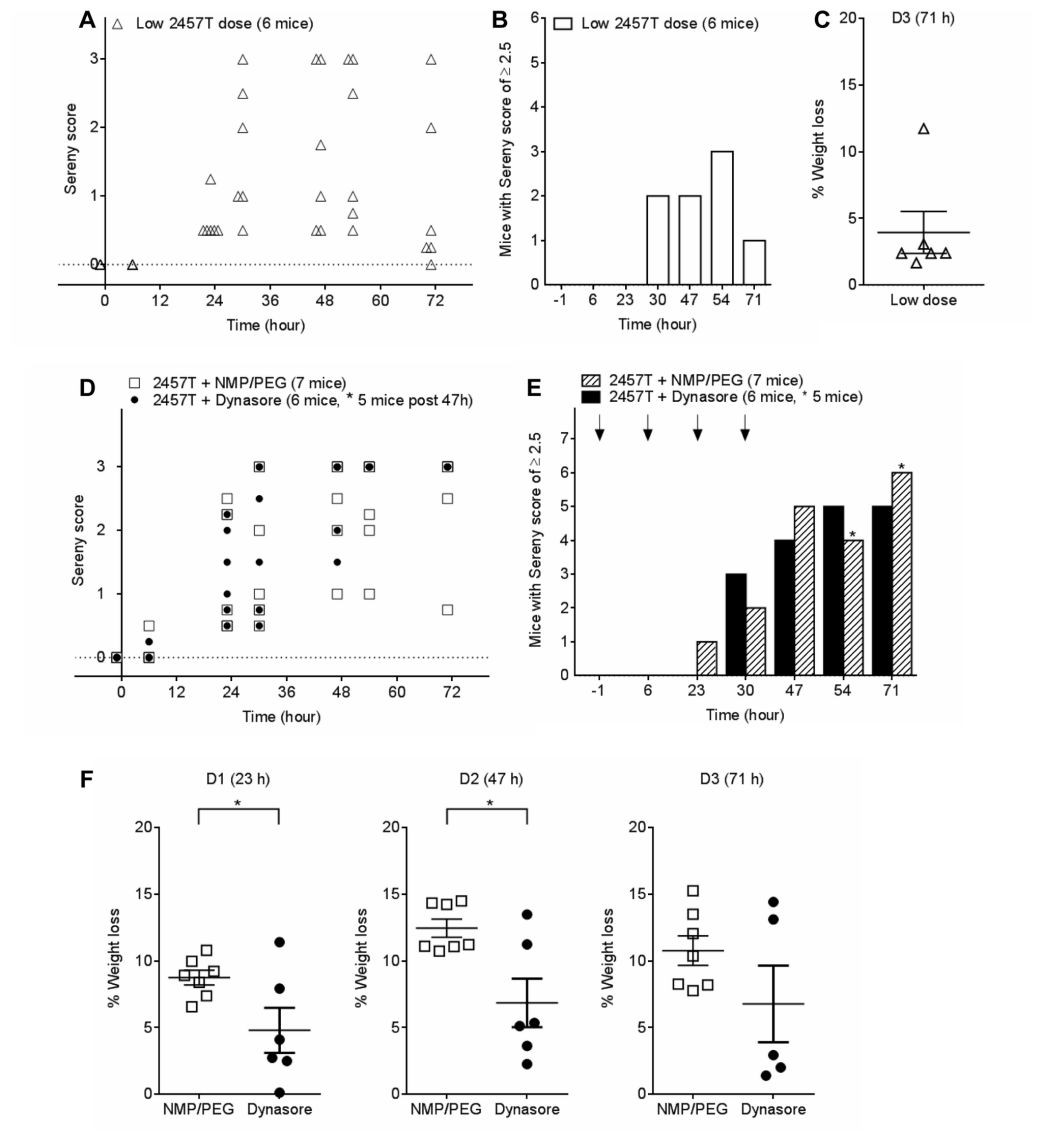
Dynasore protects mice against weight loss from *S. flexneri* 2457T ocular infection (reduced bacterial challenge). (A) Sereny scores of mice infected with 2.5 × 10^7^ CFUs *S. flexneri* 2457T from 0 - 71 h post inoculation. Each symbol represents one or more mice. (B) The total number of mice with a Sereny score of ≥ 2.5 was calculated and plotted as a bar graph for each time point. (C) The percentage weight loss for each mouse on D3 was calculated. (D) Sereny scores of mice infected with 2.5 × 10^7^ CFUs *S. flexneri* 2457T at t = 0 and IP injected with either 100 μL of 5.5 mg/mL of dynasore in NMP/PEG (30mg/kg) or 100 μL NMP/PEG at t = -1, 7, 23, 30 h post inoculation. Each symbol represents at least one mouse. (E) The total number of mice with a Sereny score of ≥ 2.5 was calculated and plotted as a bar graph for each time point. Arrows indicate injection with dynasore or NMP/PEG alone. (F) The percentage weight loss for each mouse on D1, D2 and D3 were calculated. Data are represented as mean ± SEM (Student's *t*-test, *p < 0.05).

Since dynasore treatment reduced weight loss associated with *Shigella* infection but not the extent of ocular inflammation, further studies were performed using a reduced *Shigella* dose. Mice were infected with 2.5 × 10^7^ CFUs *S. flexneri* 2457T and were weighed on D0 and D3. The mice developed inflammation at a slightly slower rate compared to the higher *Shigella* dose (Figures S5C and S5D); the inflammation peaked at 54 h with three out of six mice developing an inflammation with a score of ≥ 2.5 (Figure 7A and 7B). The mice lost an average of 4% of their body weight (Figure 7C).

In a second study with the same lower challenge dose, mice were given injection of dynasore (30 mg/kg) or vehicle at t = -1, 6, 23 and 30 h. Mice in both groups developed ocular inflammation at a similar rate and no difference in Sereny scores was observed (Figure 7D and 7E). Similar to the high dose infection, dynasore significantly protected mice against weight loss associated with *Shigella* infection when compared to the vehicle group on D1 (4.81 ± 1.68% vs. 8.75 ± 0.55%, *p < 0.05) and D2 (6.85 ± 1.82% vs. 12.46 ± 0.67%, *p < 0.05) (Figure 7F). The group of mice in this study had ruffled fur and displayed reduced mobility similar to the mice in the higher challenge dose which were give dynasore or vehicle injection.

In the ocular infection model, dynasore afforded significant protection against weight loss but did not reduce ocular inflammation. In contrast, mice infected with *∆icsA* (RMA2041) had similar weight loss to 2457T but no ocular inflammation was observed (Figure S5C - S5E), suggesting that dynasore is not preventing *S. flexneri* cell to cell spreading *in vivo.*

### Effect of dynasore on *S. flexneri*-induced HeLa cell death

Since dynasore did not seem to affect *S. flexneri* cell to cell spreading and ameliorate inflammation in vivo, we sought to determine if dynasore could be targeting TTSS effectors encoded on the VP [56], thus reducing the cytotoxic effects of the TTSS effectors, using an *in vitro* cytotoxicity assay. Previously IpaB and MxiA, both TTSS effectors, have been reported to play a role in weight loss during *S. flexneri* infection [56,57]. HeLa cells were infected with WT, *∆icsA* or VP¯ *S. flexneri* strains and LDH release was measured (Figure S6). HeLa cells infected with the VP¯ had very little cell death (2.34 ± 0.53%), comparable to the uninfected HeLa cells (5.78 ± 2.99%). In contrast, HeLa cells infected with WT and *∆icsA* strains had 40.2 ± 2.37% and 42.04 ± 2.79% cell death, respectively. Therefore in the absence of *icsA*, the bacteria still retained significant cytotoxicity presumable due the presence of TTSS effectors encoded by the VP. The weight loss observed mice infected with the *∆icsA* strain in this study (Figure S5) could be partly attributed to the cytotoxicity from the TTSS effectors. 

**Figure 8:**
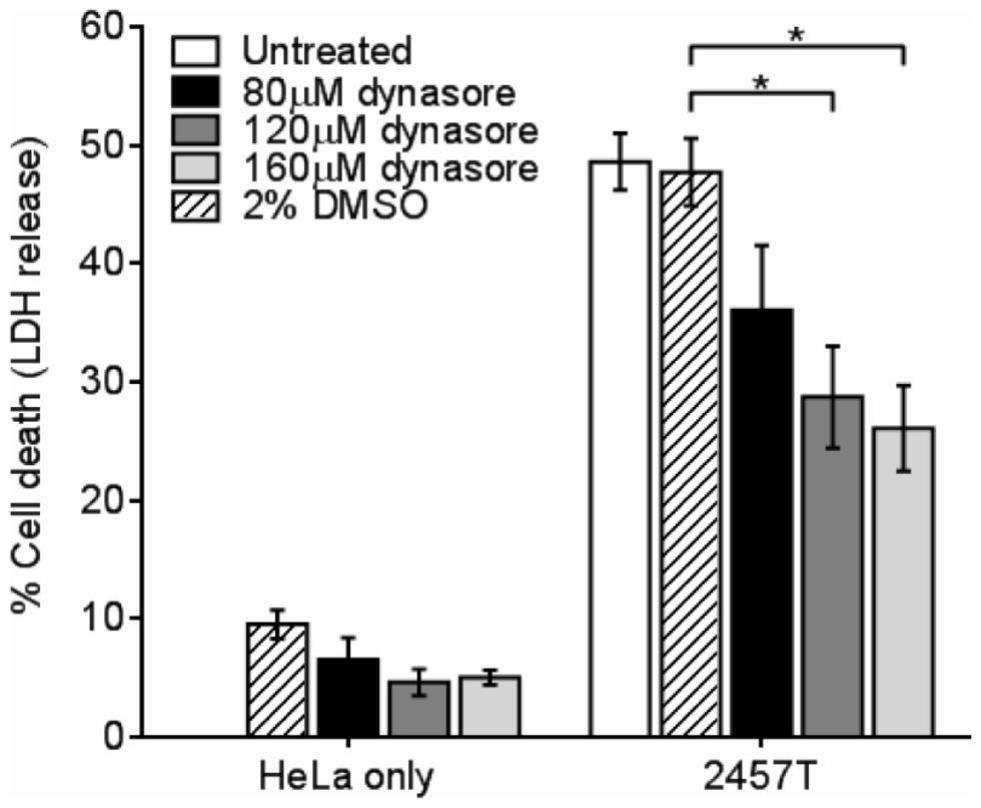
Dynasore reduces HeLa cell death during *S. flexneri* 2457T infection. HeLa cells were infected with *S. flexneri* 2457T in a 96-well tray as described in the Methods. LDH release was measured in the presence of dynasore or DMSO. Data are represented as mean ± SEM of independent experiments (n = 3), analysed with one-way ANOVA (*p* = 0.0043), followed by Tukey's post hoc test (*p < 0.05). No differences in LDH release were observed in the presence of dynasore or DMSO in the absence of bacterial infection.

Next we treated HeLa cells with increasing concentrations of dynasore or with the DMSO vehicle alone (Figure 8). No differences in LDH release was observed between dynasore and DMSO in uninfected HeLa cells. *S. flexneri*-induced HeLa cytotoxicity was significantly reduced in the presence of 120 μM (28.72 ± 4.30%) and 160 μM dynasore (26.1 ± 3.63%) compared to DMSO-treated cells (47.73 ± 2.84%, *p < 0.05), suggesting dynasore reduces *S. flexneri* induced cytotoxicity *in vitro* and may explain the weight loss protection observed *in vivo*.

## Discussion

*Shigella* infection remains a significant problem in developing countries. In this study we have identified a potential intervention strategy by targeting dynamin II, a key component of the clathrin mediated pathway. Previously dynamin II was shown to be important for *Shigella* engulfment, but not protrusion into the neighbouring cell [24]. In this study the role of dynamin II in facilitating *Shigella* cell spreading was explored with a well described dynamin II inhibitor, dynasore, as well as by siRNA knockdown. 

Inhibition of the dynamin II GTPase activity with dynasore as well as depletion of dynamin II with siRNA knockdown significantly reduced *S. flexneri* plaque size. A reduction in plaque formation could be attributed to a lack of protrusion formation. However *S. flexneri* protrusion formation was not affected by dynasore, which suggests that dynamin II and hence endocytosis is critical for *S. flexneri* entry into neighbouring cell and not exit from the initially infected cell. 

These results are in agreement with recently reported findings by Fukumatsu et al. [24], where the involvement of dynamin II, as well components of the of the clathrin-mediated endocytic pathway have been shown to be important for *S. flexneri* cell spreading and pseudopodia formation. Dynamin II was also reported to accumulate around bacteria containing pseudopodia [24]. 

Inhibition of dynasore reduced *Listeria* plaque formation to a lesser extent since plaque formation was still observed when HeLa cells were treated with 120 μM dynasore. At the same dynasore concentration, plaque formation by *Shigella* was abolished. These results suggest *Listeria* relies less on dynamin II during cell to cell spreading. Previously dynamin II had been shown to associate with the *Listeria* comet tail, although the role of the protein was not explored [58]. Recently Fukumatsu et. al. reported that clathrin-mediated endocytosis is not the primary pathway utilised by *Listeria* during cell to cell spreading [24]. Since dynamin II is a critical component of clathrin-mediated endocytosis, dynamin II may localise non-specifically to the *Listeria* F-actin tail or have other unexplored functions. 

*S. flexneri* cell to cell spreading is dependent on the bacteria's IcsA protein, a polarly localised outer membrane protein. IcsA is predominantly expressed on the old pole, i.e. the pole that was present before cellular division which gives rise to new daughter poles [50]. IcsA interacts with host proteins such as N-WASP, which recruits the Arp2/3 complex to initiate F-actin polymerisation, allowing the bacteria to spread intracellularly and between cell to cell [50-52]. In the absence of IcsA, F-actin tails and subsequent protrusion formation is abolished. The lack of dynamin II recruitment to the *∆icsA* mutants suggests that dynamin II associates with the F-actin tail as reported for *Listeria* [58]. The lack of dynamin II involvement in protrusion formation however, also suggests that its interaction with F-actin tail could be non-specific. Actin binding proteins such as Nck has been shown to associate non-specifically with the S. *flexneri* F-actin tail [59]. Alternately dynamin II could play a minor role in membrane remodelling. BAR domain proteins, such as syndapin and sorting nexin 9 (SNX9), interact with N-WASP and dynamin [43,60]. Double labelling with anti-N-WASP and anti-dynamin revealed that dynamin II is localised adjacent to N-WASP at the S. *flexneri* old pole. Some overlap of N-WASP and dynamin II labelling was observed. 

Mice were initially with challenged the WT, *∆icsA* and VP¯ *S. flexneri* strains to establish suitable controls. IcsA is important for *Shigella* cell spreading and absence of the protein prevented the bacteria from disseminating further as no inflammation was observed. However, the *∆icsA* strain can still invade the epithelial cells and have TTSS proteins encoded on the VP such as OspG that can interfere with the host signaling pathways to prevent clearance of the bacteria [61]. Previously it was reported that Balb/c mice inoculated intranasally with 2 × 10^7^ and 2 × 10^8^ CFUs *∆icsA* (*∆virG*) mutant lost >20% of their body weight 5 days post inoculation [57]. Similarly, we found that the *∆icsA* strain still retained significant virulence as measured by weight loss in the ocular infection model used in this study. In a rectal model, guinea pigs inoculated with 1 × 10^9^ CFUs *S. flexneri* 2a (strain 2457T and YSH6000) and *S. flexneri* 5a (M90T) lost ~20% of their body weight. No weight loss was observed in guinea pigs inoculated with 2457T VP¯ strain [62]. It was also reported that piglets that were orally challenged with 5 × 10^9^ CFUs *S. dysenteriae* type 1 expressing the Shiga toxin lost considerable amount of weight. Weight lost was not observed in piglets inoculated with the attenuated Shiga toxin negative strain [63]. Weight loss during *S. flexneri* 2a infection has also been reported in other animal models of infection [64-66]. 

Mice were challenged with low and high doses of S. flexneri 2457T and treated with dynasore to determine if this drug could delay the progression of *Shigella* infection *in vivo*. Mice infected with the low dose *S. flexneri* lost less weight compared to mice infected with the high bacterial dose. Dynasore afforded significant protection against weight loss for both challenge doses but did not reduce ocular inflammation. In comparison, mice infected with *∆icsA* (RMA2041) had similar weight loss to 2457T but no ocular inflammation was observed. Hence weight loss during *S. flexneri* infection is unrelated to cell to cell spreading. The *in vivo* data from this study is summarised in Table 2. 

**Table 2:** Summary of Sereny test. Mice were inoculated with 5 × 10^8^ CFUs bacteria where applicable.

**Strain / treatment**	**Sereny score^*a*^**	**Mean (SD) % weight loss^*b*^**	**Figure**
WT (2457T)	0.25 - 3 (D3)	6.00 (2.52) (n = 6)	S5
*∆icsA* (RMA2041)	0 (D3)	8.88 (6.37) (n= 4)	S5
VP- (RMA2159)	0 (D3)	-0.35 (1.93) (n = 4)	S5
NMP/PEG (no infection)	N/A (D3)	6.29 (0.68) (n = 6)	S1
NMP/PEG + Dynasore (no infection)	N/A (D3)	4.44 (3.44) (n = 6)	S1
2457T + NMP/PEG (Expt 2)	2.5 - 3 (D2)	12.52 (4.04) (n = 7)	6
2457T + NMP/PEG + Dynasore (Expt 2)	3 (D2)	7.72 (3.21) (n = 7)	6

^a^ Sereny scores are between 0 - 3, recorded on D2 or D3

^b^ % Mean weight loss on D2 or D3 (see Sereny scores) compared to D0

N/A Not applicable

We investigated if dynasore could be targeting or inhibiting TTSS effectors encoded on the VP. Addition of 120 μM dynasore reduced *S. flexneri*-induced HeLa cell death by ~39% compared to DMSO-treated cells. The *∆icsA* mutant strain exhibited similar cytotoxicity to the WT strain, suggesting the HeLa cell death observed is not due to cell to cell spreading. Dynasore's ability to reduced *S. flexneri*-induced HeLa cytotoxicity *in vitro* could explain the reduction in weight loss in mice, even though ocular inflammation was not decreased. Previously Balb/c mice inoculated intranasally with 2 × 10^8^ CFUs *∆ipaB* mutant had comparable body weight compared to saline-treated mice. Furthermore the *∆ipaB* mutant had no cytotoxic effects, as measured by LDH release in mouse J774 macrophages [57]. IpaB (Invasion plasmid antigen B) is a secreted TTSS protein and has recently been shown to induce cell death of macrophages by disrupting ion homeostasis within endosomal compartments leading to subsequent Caspase-1 activated cell death (pyroptosis) [67]. In a recent report adult B6 mice infected with *S. flexneri* YSH6000 lost 25% of their body weight 72 hours after initial infection. Mice infected with a *mxiA::Tn5* insertion mutant (strain S325) did not have any appreciable weight loss [64]. MxiA is a key structural protein of the TTSS and *mxiA* mutants are avirulent [64,68].

Limitations in this study include the dosage of dynasore and the drug carrier used. In the *in vivo* experiments mice were given four dynasore injections during the course of infection. It is possible that the drug did not reach an effective concentration *in vivo*. The NMP/PEG carrier did not result in significant toxicity alone but the combination of NMP/PEG and the *Shigella* infection was detrimental to the mice. This limited the number of dynasore injections that could be administered during the infection. Nonetheless these results suggest that dynamin II inhibition may be a novel intervention strategy for preventing *Shigella* dissemination. The use of a different carrier solvent, increased or prolonged dynasore dosage and a different administration route may improve the efficacy of dynasore. Dynasore also inhibits the GTPase activity of Drp1 (Dynamin-related protein 1), a mitochondrial dynamin, which regulates mitochondrial fission [34,69]. Since dynasore inhibits both dynamin II and Drp1, a role for dynasore inhibition Drp1 in preventing weight loss in mice during *S. flexneri* infection cannot be ruled out. We are currently investigating this possibility.

In conclusion, dynamin II is a critical component of *Shigella* cell to cell spreading. Our data suggest that the process of *Shigella* cell to cell spreading relies in part on components important for endocytosis. Furthermore dynamin II inhibition with dynasore or a similar compound could provide a potential intervention strategy to dampen *Shigella* dissemination. The effect of dynasore on TTSS-induced cell death is currently being investigated.

## Supporting Information

Figure S1 **1:9 NMP/PEG as a vehicle for dynasore.** (A - B) HeLa cells were infected with *S. flexneri* 2457T in a plaque assay using a 6-well tray as described in the Methods. Plaque formation was performed in the presence of dynasore dissolved in either DMSO or NMP/PEG. (A) The total plaque counts or (B) mean plaque diameters from each well from independent experiments were calculated. Data are represented as mean ± SEM of independent experiments (n = 3), analysed with one-way ANOVA (*p* < 0.0001), followed by Tukey's post hoc test (*p < 0.05, **p < 0.01, ***p < 0.001, ****p < 0.0001). (C - D) Mice were not adversely affected by IP injection with either 100 μL NMP/PEG or 100 μL 5.5 mg/mL of dynasore in NMP/PEG (30mg/kg) at t = 0, 7, 24, 31 h in the absence of bacterial inoculation. Each symbol represents one mouse. Data are represented as mean ± SEM, analysed with one-way ANOVA (*p* = 0.0241 for NMP/PEG and *p* = 0.4529 for dynasore in NMP/PEG). Tukey's post hoc test was carried out for NMP/PEG (*p < 0.05).
(TIF)

Figure S2 **Intracellular growth of *S. flexneri* 2457T in HeLa cells is not affected by dynasore.** HeLa cells were infected with *S. flexneri* 2457T for 1 h in a 24-well tray. HeLa cells were washed thrice with D-PBS and incubated with MEM containing 40 µg/mL of gentamicin (t=0) to exclude extracellular bacteria. Concurrently HeLa cells were treated with 80 μM dynasore or DMSO. For each condition, two wells were prepared for each time point (t =1, 2, 4 and 6 h). At each interval, HeLa cells were washed, followed by lysis with 0.1% Triton-X 100 to recover intracellular bacteria. Data are represented as mean from three independent experiments.
(TIF)

Figure S3 ***S. flexneri* 2457T entry into HeLa cells is not affected by dynasore.** HeLa cells were infected with *S. flexneri* 2457T for 1 h in a 24-well tray. Concurrently HeLa cells were treated with 80 μM dynasore or DMSO. For each condition, two wells were prepared. After the 1 h invasion, HeLa cells were washed thrice with D-PBS and incubated with MEM containing 40 µg/mL of gentamicin to exclude extracellular bacteria. After 2 h, HeLa cells were washed, followed by lysis with 0.1% Triton-X 100 to recover intracellular bacteria. Data are represented as mean from three independent experiments.
(TIF)

Figure S4 **Dynamin II is localised adjacent to N-WASP at *S. flexneri* 2457T pole.** HeLa cells were infected with *S. flexneri* 2457T in an invasion assay as described in the Methods. Bacteria and HeLa nuclei were stained with DAPI (blue), N-WASP was stained with anti-N-WASP and Alexa Fluor 488-conjugated secondary antibody (green); and dynamin II was stained with anti-dynamin and Alexa Fluor 594-conjugated secondary antibody (red). Images were taken at 100× magnification. Scale bar = 10 μm. (A) – (C) The white arrows indicate protrusions. Insert shows 2× enlargement of the indicated region. Bacteria are outlined with white dotted lines. (D) The bacteria indicated with the white arrow are enlarged 6×. The thin arrowhead points to N-WASP and the thick arrowhead points to dynamin II. The yellow arrows point to areas of N-WASP and dynamin II overlap. The experiment was repeated twice and representative images are shown.
(TIF)

Figure S5 **Establishment of a mouse Sereny test to measure keratoconjuctivitis caused by *S. flexneri*** **2457T**. (A) The left eye was inoculated with LB broth (control) and the right eye was inoculated with 5 × 10^8^ CFUs WT *S. flexneri* 2457T. Mouse keratoconjuctival inflammation was defined as follows: a score of 1 is defined as mild keratoconjuctivitis where the eye lid is slightly swollen; a score of 2 is defined as severe keratoconjuctivitis where the eye is half closed and a score of 3 is defined as fully developed keratoconjunctivitis where the eye is completely closed. (B) Histology of fornix (*F*), palpebral conjunctiva (*C*) and cornea (*Cor*) of the control eye (left - LB) and *S. flexneri* infected eye with a Sereny score of 3 (right - 2457T). Polymorphonuclear leukocytes are infiltrating into the epithelial layer of the fornix (◄) and submucosal area of the conjuctiva and cornea (←) (60× magnification). (C-E) Mouse Sereny test with 5 × 10^8^ CFUs *S. flexneri* 2457T, *∆icsA* (RMA2041) and virulence plasmid negative strain (VP¯) (RMA2159). (C) Sereny scores of mice infected with *S. flexneri* 2457T, RMA2041 and RMA2159 from 0 - 71 h post infection. Each symbol represents one mouse. (D) The total number of mice with a Sereny score of ≥ 2.5 was calculated and plotted as a bar graph for each time point. (E) The percentage weight loss for each mouse on D3 was calculated. Data are represented as mean ± SEM, analysed with one-way ANOVA (*p* = 0.0171), followed by Dunnett's post hoc test with comparison to the mean of the VP¯ strain (*p < 0.05).
(TIF)

Figure S6 **HeLa cell death during *S. flexneri* 2457T and *∆icsA* infection.** HeLa cells were infected with *S. flexneri* 2457T, *∆icsA* (RMA2041) and virulence plasmid negative strain (VP¯) (RMA2159) in a 96-well tray and LDH release was measured as described in the Methods. Data are represented as mean ± SEM of independent experiments (n = 3), analysed with one-way ANOVA (*p* < 0.0001), followed by Tukey's post hoc test (****p < 0.0001).
(TIF)
