# Supplementary figures and images for: Impact of Dynasore an Inhibitor of Dynamin II on Shigella flexneri Infection
Source: PLoS One. 2013 Dec 19;8(12):e84975. doi: 10.1371/journal.pone.0084975 (PMC3868620; doi:10.1371/journal.pone.0084975)

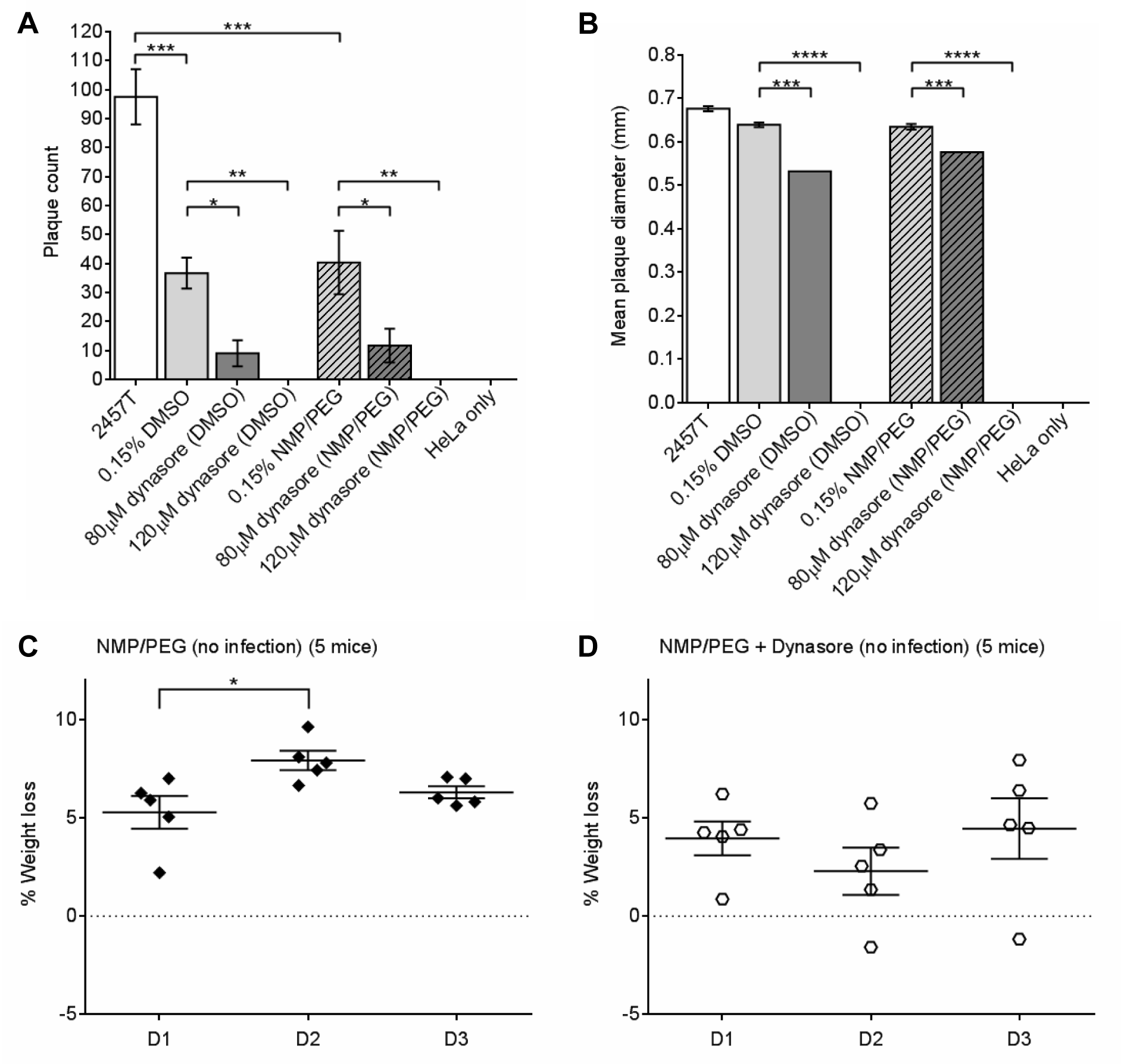

Supplement: Figure S1 — 1:9 NMP/PEG as a vehicle for dynasore. (A - B) HeLa cells were infected with S. flexneri 2457T in a plaque assay using a 6-well tray as described in the Methods. Plaque formation was performed in the presence of dynasore dissolved in either DMSO or NMP/PEG. (A) The total plaque counts or (B) mean plaque diameters from each well from independent experiments were calculated. Data are represented as mean ± SEM of independent experiments (n = 3), analysed with one-way ANOVA (p < 0.0001), followed by Tukey's post hoc test (*p < 0.05, **p < 0.01, ***p < 0.001, ****p < 0.0001). (C - D) Mice were not adversely affected by IP injection with either 100 μL NMP/PEG or 100 μL 5.5 mg/mL of dynasore in NMP/PEG (30mg/kg) at t = 0, 7, 24, 31 h in the absence of bacterial inoculation. Each symbol represents one mouse. Data are represented as mean ± SEM, analysed with one-way ANOVA (p = 0.0241 for NMP/PEG and p = 0.4529 for dynasore in NMP/PEG). Tukey's post hoc test was carried out for NMP/PEG (*p < 0.05). (TIF) [file pone.0084975.s001.tif]

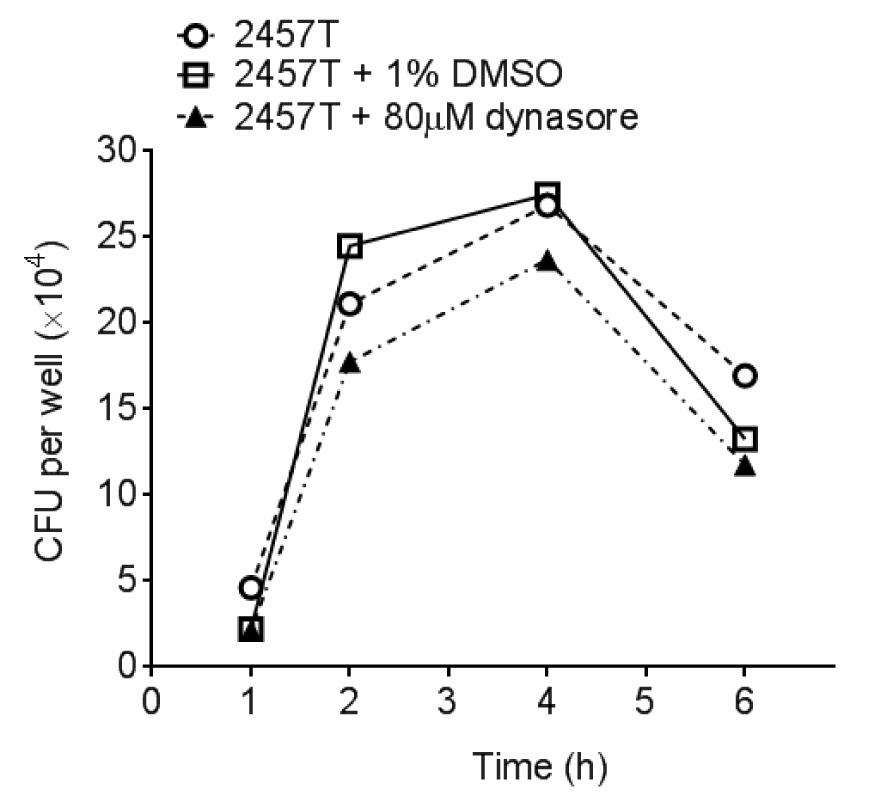

Supplement: Figure S2 — Intracellular growth of S. flexneri 2457T in HeLa cells is not affected by dynasore. HeLa cells were infected with S. flexneri 2457T for 1 h in a 24-well tray. HeLa cells were washed thrice with D-PBS and incubated with MEM containing 40 µg/mL of gentamicin (t=0) to exclude extracellular bacteria. Concurrently HeLa cells were treated with 80 μM dynasore or DMSO. For each condition, two wells were prepared for each time point (t =1, 2, 4 and 6 h). At each interval, HeLa cells were washed, followed by lysis with 0.1% Triton-X 100 to recover intracellular bacteria. Data are represented as mean from three independent experiments. (TIF) [file pone.0084975.s002.tif]

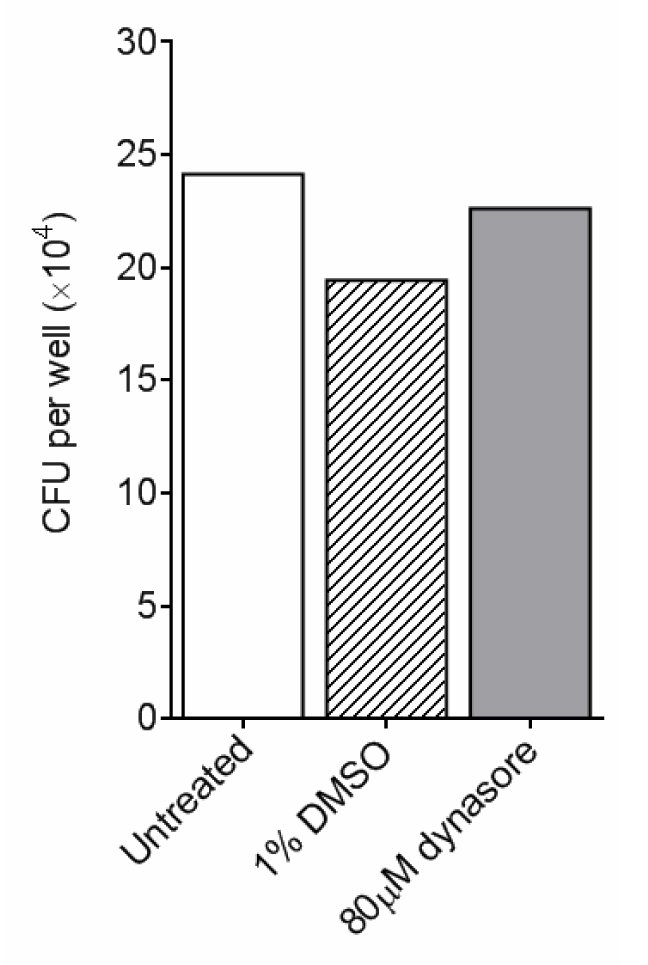

Supplement: Figure S3 — S. flexneri 2457T entry into HeLa cells is not affected by dynasore. HeLa cells were infected with S. flexneri 2457T for 1 h in a 24-well tray. Concurrently HeLa cells were treated with 80 μM dynasore or DMSO. For each condition, two wells were prepared. After the 1 h invasion, HeLa cells were washed thrice with D-PBS and incubated with MEM containing 40 µg/mL of gentamicin to exclude extracellular bacteria. After 2 h, HeLa cells were washed, followed by lysis with 0.1% Triton-X 100 to recover intracellular bacteria. Data are represented as mean from three independent experiments. (TIF) [file pone.0084975.s003.tif]

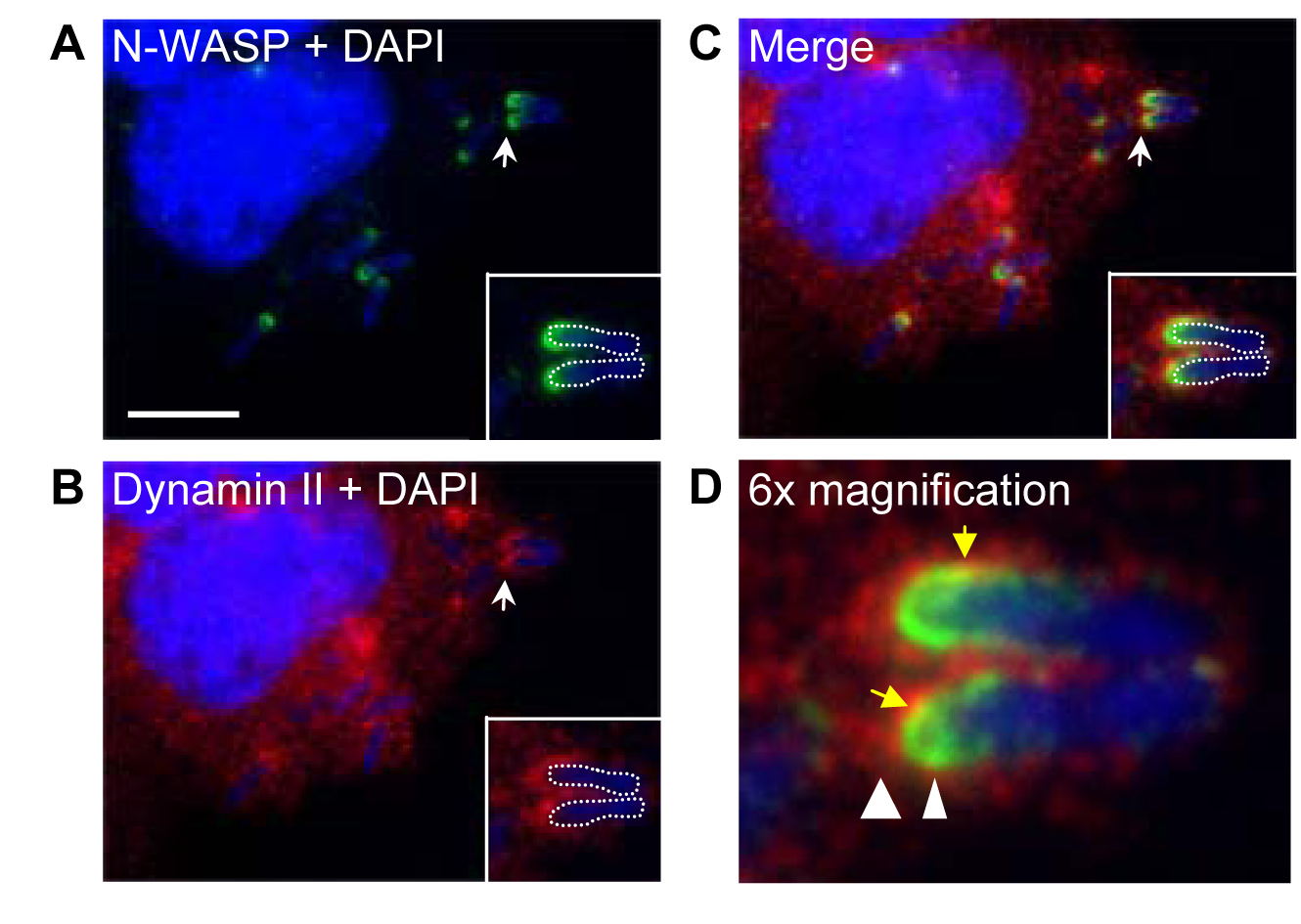

Supplement: Figure S4 — Dynamin II is localised adjacent to N-WASP at S. flexneri 2457T pole. HeLa cells were infected with S. flexneri 2457T in an invasion assay as described in the Methods. Bacteria and HeLa nuclei were stained with DAPI (blue), N-WASP was stained with anti-N-WASP and Alexa Fluor 488-conjugated secondary antibody (green); and dynamin II was stained with anti-dynamin and Alexa Fluor 594-conjugated secondary antibody (red). Images were taken at 100× magnification. Scale bar = 10 μm. (A) – (C) The white arrows indicate protrusions. Insert shows 2× enlargement of the indicated region. Bacteria are outlined with white dotted lines. (D) The bacteria indicated with the white arrow are enlarged 6×. The thin arrowhead points to N-WASP and the thick arrowhead points to dynamin II. The yellow arrows point to areas of N-WASP and dynamin II overlap. The experiment was repeated twice and representative images are shown. (TIF) [file pone.0084975.s004.tif]

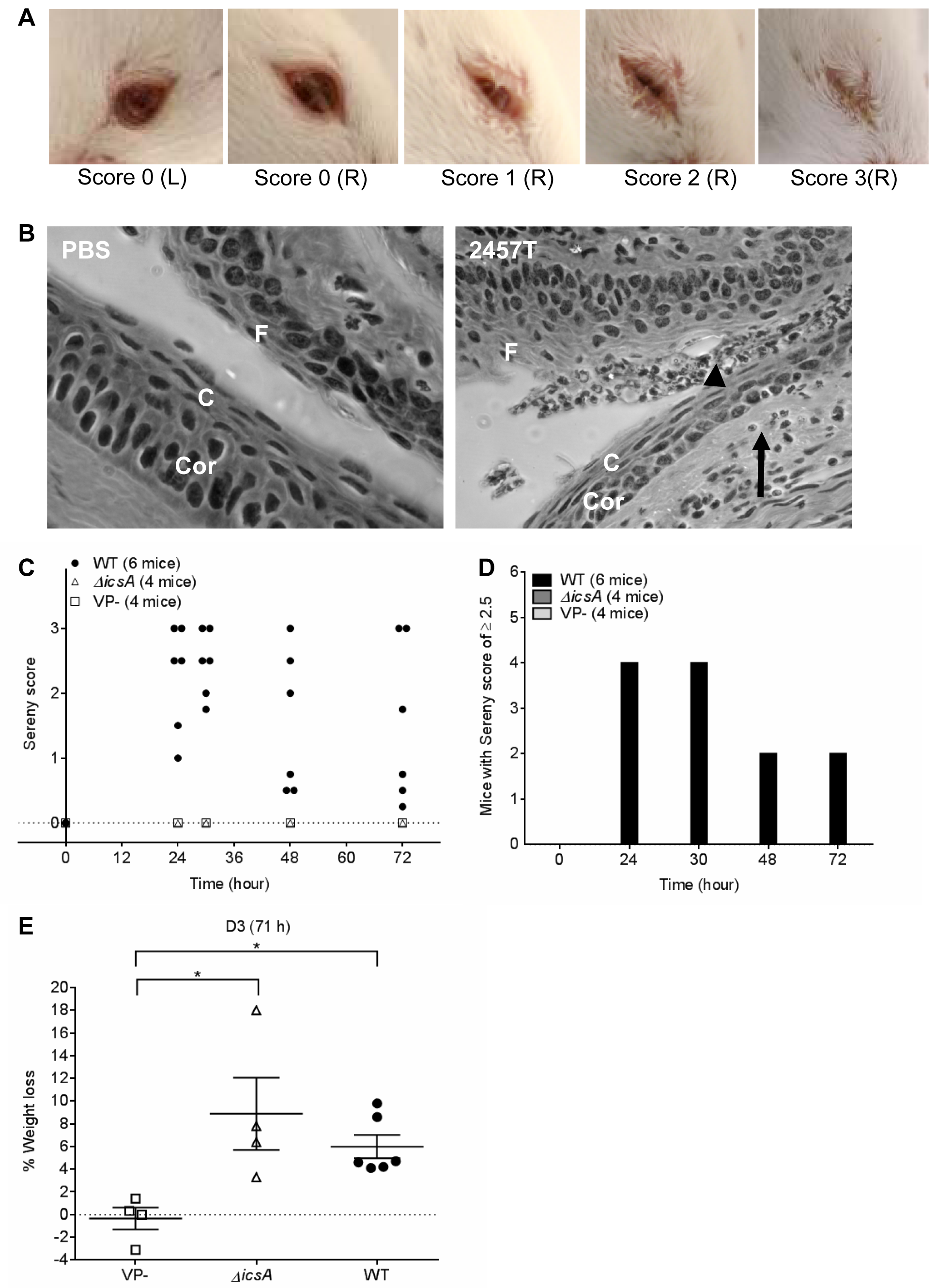

Supplement: Figure S5 — Establishment of a mouse Sereny test to measure keratoconjuctivitis caused by S. flexneri 2457T. (A) The left eye was inoculated with LB broth (control) and the right eye was inoculated with 5 × 10^8^ CFUs WT S. flexneri 2457T. Mouse keratoconjuctival inflammation was defined as follows: a score of 1 is defined as mild keratoconjuctivitis where the eye lid is slightly swollen; a score of 2 is defined as severe keratoconjuctivitis where the eye is half closed and a score of 3 is defined as fully developed keratoconjunctivitis where the eye is completely closed. (B) Histology of fornix (F), palpebral conjunctiva (C) and cornea (Cor) of the control eye (left - LB) and S. flexneri infected eye with a Sereny score of 3 (right - 2457T). Polymorphonuclear leukocytes are infiltrating into the epithelial layer of the fornix (◄) and submucosal area of the conjuctiva and cornea (←) (60× magnification). (C-E) Mouse Sereny test with 5 × 10^8^ CFUs S. flexneri 2457T, ∆icsA (RMA2041) and virulence plasmid negative strain (VP¯) (RMA2159). (C) Sereny scores of mice infected with S. flexneri 2457T, RMA2041 and RMA2159 from 0 - 71 h post infection. Each symbol represents one mouse. (D) The total number of mice with a Sereny score of ≥ 2.5 was calculated and plotted as a bar graph for each time point. (E) The percentage weight loss for each mouse on D3 was calculated. Data are represented as mean ± SEM, analysed with one-way ANOVA (p = 0.0171), followed by Dunnett's post hoc test with comparison to the mean of the VP¯ strain (*p < 0.05). (TIF) [file pone.0084975.s005.tif]

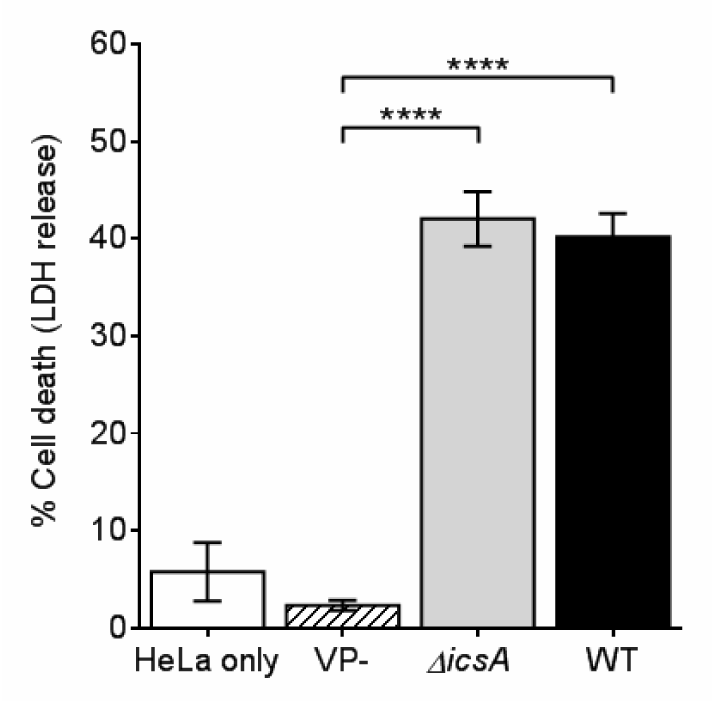

Supplement: Figure S6 — HeLa cell death during S. flexneri 2457T and ∆icsA infection. HeLa cells were infected with S. flexneri 2457T, ∆icsA (RMA2041) and virulence plasmid negative strain (VP¯) (RMA2159) in a 96-well tray and LDH release was measured as described in the Methods. Data are represented as mean ± SEM of independent experiments (n = 3), analysed with one-way ANOVA (p < 0.0001), followed by Tukey's post hoc test (****p < 0.0001). (TIF) [file pone.0084975.s006.tif]
